# Breast Milk Intake from 1 to 8.5 Months of Lactation in the Multisite Mothers, Infants and Lactation Quality (MILQ) Study

**DOI:** 10.1016/j.advnut.2025.100456

**Published:** 2025-08-26

**Authors:** Sophie E Moore, Sarita Devi, Anura Kurpad, Janet M Peerson, Sophie Hilario Christensen, Md Munirul Islam, Gilberto Kac, Kim F Michaelsen, Gabriela Torres Silva, Lindsay H Allen, The MILQ Study Consortium, Lindsay H Allen, Sophie E Moore, Gilberto Kac, Kim F Michaelsen, Christian Mølgaard, M Munirul Islam, Maria Andersson, Setareh Shahab-Ferdows, Sophie H Christensen, Jack I Lewis, Janet M Peerson, Xiuping Tan, Daphna K Dror, Andrew M Doel, Daniela de Barros Mucci, Bruna C Schneider, Farhana Khanam, Adriana Divina de Souza Campos, Gabriela Torres Silva, Fanta Nije, Mehedi Hassan, Amanda C Figueiredo, Daniela Hampel

**Affiliations:** 1 Department of Women & Children’s Health, King’s College London, London, United Kingdom; 2 MRC Unit The Gambia at the London School of Hygiene and Tropical Medicine, Fajara, The Gambia; 3 Division of Nutrition, St. John's Research Institute, St. John's National Academy of Health Sciences, Bangalore, India; 4 Department of Physiology, St. John's Medical College, St. John's National Academy of Health Sciences, Bangalore, India; 5 USDA, ARS Western Human Nutrition Research Center, University of California Davis, Davis, California, United States; 6 Department of Nutrition, Exercise and Sports, Faculty of Science, University of Copenhagen, Copenhagen, Denmark; 7 Center for Clinical Research and Prevention, Copenhagen University Hospital - Bispebjerg and Frederiksberg Hospital, Copenhagen, Denmark; 8 Nutrition Research Division, icddr,b, Dhaka, Bangladesh; 9 Nutritional Epidemiology Observatory, Josué de Castro Nutrition Institute, Federal University of Rio de Janeiro, Rio de Janeiro, RJ, Brazil; 10 Institute for Global Nutrition, Department of Nutrition, University of California, Davis, California, United States

**Keywords:** exclusive breastfeeding, lactation, human milk, nonmilk oral intake, milk volume, deuterium oxide, stable isotope, dose-to-mother

## Abstract

Human milk from healthy, well-nourished women is the optimal nutrition for infants and young children, and exclusive breastfeeding is recommended for the first 6 mo. In the context of poor diets, the quality of milk may be compromised, and understanding the relationships between maternal diet, human milk nutrient concentrations, and milk intakes in young infants is important for guiding policy. The Mothers, Infants, and Lactation Quality (MILQ) study collected human milk samples from 1 to 8.5 mo of lactation in 558 well-nourished but unsupplemented women in Bangladesh, Brazil, Denmark, and The Gambia. Milk intakes were measured at 3 visits postnatally (1–3.49 mo, 3.5–5.99 mo, and 6.0–8.5 mo). Milk intakes were assessed in 3 sites (Bangladesh, Brazil, and The Gambia) using the stable isotope dilution dose-to-mother method. In Denmark, intakes were measured by test weighing, and volume data were corrected by a factor of 1.05 to account for insensible water losses. The mean ± standard deviation human milk intake across sites and time points was 781 ± 193 g/d. Milk intakes were comparable among sites early in lactation, but as intakes decreased across lactation, between-site differences emerged as nonmilk feeds were introduced. Exclusively breastfed infants consumed greater volumes of milk each day than mixed-fed infants. When expressed against infant body weight, a gradual decrease in milk intake was observed across lactation. Milk nutrient concentrations were largely unrelated to daily milk intakes. Therefore, correlations between milk nutrient concentrations and total daily intake of nutrients were mostly positive and strong. Mean milk intake in the MILQ study was consistent with previously published global data, although variability was observed across lactation and between contexts. Infants consuming greater milk volumes had greater daily intakes of milk nutrients. The implications for infant status and recommended nutrient intakes require further investigation.

**Statement of significance**

This article in the series providing reference values for nutrients in human milk describes the milk volumes produced by lactating mothers and consumed by their infants across the first 8.5 mo of lactation. To our knowledge, this is the first study to measure milk volumes at the same time as assessing the concentrations of multiple nutrients at repeated time points during lactation, enabling calculation of the actual intakes of nutrients consumed and informing infant nutrient intake recommendations. It has also revealed, for the first time, that milk volumes do not independently affect nutrient concentrations, so, in addition to milk nutrient concentration, usual volume impacts infant intake.

## Introduction

Exclusive breastfeeding is recommended for the first 6 mo of life [1], and it is assumed that human milk from healthy, well-nourished women provides adequate amounts of most nutrients to support optimal growth and development of infants. However, the volume of milk consumed by exclusively breastfed (EBF) infants varies widely [2,3], and the impact this has on nutrient intakes in infants is underresearched. The multicenter Mothers, Infants, and Lactation Quality (MILQ) study was established to develop reference values (RVs) for human milk nutrients across lactation [4]. The key objectives of the MILQ study were to consider how intakes of each nutrient vary according to milk volume and to evaluate the broader implications for recommended nutrient intakes in infants.

Assessment of human milk intake is challenging as, unlike assessment of dietary intake, the amount consumed is not directly observable. A number of methods for measuring intake have been developed and applied, including test weighing and maternal milk expression. However, both of these methods are subject to error as the processes involved may interfere with usual behaviors and, therefore, results obtained may not reflect habitual intakes [5,6]. Further, because of the heavy burden on participating families, intakes are often restricted to a limited period (often <5 d) and, therefore, are subject to error from day-to-day variation. To counter these challenges, Coward et al. [5] developed a stable isotope tracer method (the “dose-to-mother” method) to measure human milk (and nonmilk) oral intakes. In this method, the mother receives an oral dose of deuterium labeled water (^2^H_2_O), and the disappearance of the isotope from her total body water (sampled from saliva or urine) is monitored over a 14-d period while, at the same time, monitoring the appearance of the tracer in, and its subsequent disappearance from, the infant’s body water (again, measured in serial urine or saliva samples). This method has been successfully employed across multiple studies and in contrasting settings and has the advantages that it is easier to apply in field settings and, in larger population groups, monitors intakes over a 14-d period, and is less impactful on usual behaviors.

A systematic review and meta-analysis published in 2023 found the mean daily human milk intake from 167 global studies to be 690 g (670 mL; 121 g [117 mL]/kg/d) [7]. However, this value represents intakes for infants and young children from 0 to 35.9 mo and includes mixed-fed infants where the proportion of milk derived from human milk is varied. Among the subset of EBF infants, mean milk intakes were 706 g/d (685 mL/d) from 0 to 2.9 mo, 781 g/d (758 mL/d) from 3 to 5.9 mo, and 756 g/d (734 mL/d) from 6 to 11.9 mo (to provide direct comparability between data presented from different sources, data presented in mL/d has been converted into g/d using a conversion factor of 1.03) [7,8]. Published data from 12 countries using the dose-to-mother method in infants aged 0 to 24 mo observed a mean milk intake of 780 g/d (757 mL/d) [9]; the value consistently used as the global estimate of daily intakes. Data from a community-based study in Pelotas, Brazil, using the dose-to-mother method found the mean intake of human milk at 4 mo of age to be 806 g/d (783 mL/d) in EBF infants compared with 778 g/d (755 mL/d) in partially breastfed infants [10]. Similarly, in a cross-sectional study of infants from rural Bangladesh, also using the stable isotope dilution “dose-to-mother” method, intakes in EBF infants were 911 g/d (884 mL/d) and in non-EBF infants 815 g (791 mL/d) [9]. Of note, the range of milk intakes among the EBF group (*N* = 73 infants) ranged from 543 to 1273 g/d (527–1236 mL/d) [11]. Such a variation is consistent with the broader literature [7].

In this series of initial papers from the MILQ study, RVs for milk nutrients have been presented [[12], [13], [14], [15]]. What is apparent is that both between and within sites, there is a variation in nutrient concentrations despite the study purposefully recruiting healthy, well-nourished women. As highlighted above, there is also interindividual variation in daily milk intakes, even among EBF young infants. However, what is not understood is whether milk concentrations of nutrients vary in response to habitual patterns in consumption, thereby “harmonizing” nutrient intakes in infants. The objectives of this paper are to, first, describe patterns in human milk intake from 1 to 8.5 mo postpartum from 4 cohorts globally and, second, relate milk intakes to measured nutrient concentrations.

## Methods

### Study design and setting

The MILQ study collected data and samples from a target of *N* = 250 mother–infant dyads in each of the 4 participating countries: Bangladesh, Brazil, Denmark, and The Gambia. These countries were selected to represent different geographic areas globally but with limited national micronutrient fortification or supplementation programs. The full research protocol for MILQ and demographic tables for all participating mothers and infants are published elsewhere [4], and details relevant to the current analysis are described below. To ensure that participants were well-nourished, relevant inclusion criteria were maternal BMI 18.5–29.9 kg/m^2^ prepregnancy (Brazil and Denmark) or <2 wk postpartum (Bangladesh and The Gambia); midupper arm circumference 23–33 cm (The Gambia) or 21–33 cm (Bangladesh); height ≥150 cm (>145 cm in Bangladesh); hemoglobin ≥100 g/L (not verified in Denmark); and nonvegan or macrobiotic diet. In addition, maternal diet quality was assessed at screening using a dietary diversity questionnaire [16]. The MILQ study protocol is published at ClinicalTrials.gov, NCT03254329. Data on milk volumes in the early postnatal period (<1 mo) were collected as part of the parallel Early-MILQ (E-MILQ) study but, because of methodological differences (e.g., data were not collected in Brazil due to post-COVID-19 restrictions and Denmark switched methods from test weighing to isotope dilution) and the different time frame for the collection of E-MILQ samples, these data will not be presented in this manuscript and will be considered separately.

At each MILQ site, participants were recruited prenatally and, following delivery, were seen at 3 postnatal time points: 1.0–3.49 mo (M1), 3.5–5.99 mo (M2), and 6.0–8.5 mo (M3). As the main purpose of MILQ was to develop nutrient RVs, each participating mother–infant dyad was randomly assigned to have their samples collected at a specific time during each visit window (e.g., if they were seen at the start of the M1 window, they were also seen at the start of the time windows for M2 and M3). This process ensured that *1*) across each country cohort, data and samples were available throughout the whole period to enable the smoothing of reference curves, and *2*) the time period between each visit for individual dyads was the same, minimizing participant burden.

At each postpartum visit, human milk intake was assessed in participating infants. In 3 sites (Bangladesh, Brazil, and The Gambia), intake was assessed during a 14-d period around the time of milk sampling, using the stable isotope dilution “dose-to-mother” method. In Denmark, intakes were measured by 24-h before- and after-feed test weighing. The decision to utilize test weighing for 24 h to measure milk intake in Denmark was made before study implementation due to concerns about Danish participants rejecting the isotopic method.

Full ethical approval was obtained at each site from the relevant ethics committee/review board [4]. Additional approval for the full study was obtained from the Institutional Review Board of the University of California, Davis, CA, United States.

### Sample collection

*Stable isotope dilution dose-to-mother method*: At each postnatal time point, milk intakes were assessed using a validated protocol that measures milk and nonmilk intakes across a 14-d period [17]. At baseline (Day 0), a sample of saliva was collected from each mother and her infant using standard procedures, which involved using an absorbent cotton swab placed in the participant’s mouth to absorb saliva, after which the saliva was squeezed out using a sterile syringe. Following sample collection, mothers were asked to consume a 30 g oral dose of deuterium oxide (^2^H_2_O, 99.8%, Cambridge Isotope Laboratories Inc), measured to the nearest 0.01 g. Subsequent saliva samples were collected from each mother and infant on days 1, 2, 3, 4, 13, and 14 postdosing. Following collection, samples were stored frozen and shipped to St. John’s Research Institute, Bangalore, India, for analysis.

The ^2^H enrichment in the collected saliva samples was measured by Fourier transform infrared spectroscopy (Agilent Technologies). Sample processing and analysis followed the Standard Operating Procedure set out by the International Atomic Energy Agency [18]. In brief, following sample thawing and centrifugation, a 30 μl aliquot of the supernatant was used for analysis after air background correction. Each sample was measured in duplicate, and the mean value was used for calculations. Before saliva measurement, the ^2^H_2_O standards prepared by dilution with deionized water at two levels (0 and 1000 mg/kg excess deuterium) were analyzed. The Fourier transform infrared spectroscopy covers the midinfrared range (5000 cm^-1^ to 500 cm^-1^). Deuterium oxide spectra were measured at 2500 cm^-1^. The human milk and nonmilk oral intake (NMOI) values were calculated by fitting the isotopic enrichment data to a two-compartment mathematical model for water turnover in the mother–infant dyad for the transfer of deuterium from mother to the infant, based on assumptions as described earlier [18].

*Test weighing*: In Denmark, human milk intake was estimated through the 24-h test weighing method [6]. In brief, at each postnatal visit, caregivers were provided with a set of digital infant scales (ADE M101000-01; ADE GmbH & Co) and asked to weigh and record the weight of their infant before and after each feed over a 24-h period. Weights were recorded to an accuracy of 5 g for weights <10 kg and 10 g for weights >10 kg, and measurements were made between the hours of 08:00 and 07:59, plus one extra weighing to define the 24 h. The mother was instructed to weigh the infant with the same clothes and diaper at paired weighings (before and after each feed) in order to get a correct estimate of milk intake defined by the weight difference. The mother logged the time and infant weight before and after the feed and which breast was used. Logs were double-entered electronically by study staff. Single feeds >400 g were regarded as being unrealistically high and marked as missing. Logs with >3 missing feedings were regarded as insufficient and were discarded. For participants with ≤3 missing feeds, missing data were imputed using hot deck imputation based on neighboring weights from the same participant.

### Milk nutrient concentrations

Concentrations of nutrients in milk were measured at each sampling time point. Details on the measurement methods employed, alongside the RVs for each nutrient, are presented in separate papers as part of this series [[12], [13], [14], [15]].

### Data analysis

Following sample analysis, all data were returned to the central MILQ database, and obvious visual outliers were removed (see below). The stable isotope dilution “dose-to-mother” method produces estimates of human milk intake and NMOI in kg/day. This value was multiplied by 1000 to express quantities as g/day. The test weighing method is thought to underestimate milk intake by ∼5% due to insensible water loss. Data from Denmark were therefore corrected by a factor of 1.05 for consistency [19].

A total of 82 observations considered invalid were initially removed by the analytical laboratory. Reasons for removal included insufficient saliva samples across the collection window to generate a reliable curve estimate. On receipt of the data, the research team removed data points considered as extreme and, therefore, unreliable (human milk intake >3000 g/d or NMOI <−4000 or >12,000 g/d).

Previous studies using the deuterium dilution method report negative values for nonmilk oral intakes. These values arise because, in infants with no water intake from nonbreast milk sources, random methodological error will produce both negative and positive estimates of NMOI around a zero mean [11].

Milk volume and NMOI values were then removed for any one of the following reasons, supported by published literature:

1.) Milk volume was not between 500 and 1300 g/d for EBF infants (reported) at visits M1 and M2.
2.) Milk volume was not between 100 and 1300 g/d for non-EBF infants (reported) at visits M1 and M2 or any child at visit M3.
3.) NMOI was not between −100 and 1300 g/d (no NMOI for Denmark).
4.) The sum of human milk volume and NMOI was not between 500 and 1300 g/d.
5.) NMOI was >250 g/d for EBF children (reported) at visits M1 and M2.

All descriptive analyses are presented using only the data from participants who remained in the dataset following the implementation of the cleaning protocol described above. To illustrate human milk intake, nonmilk oral intake, and combined human milk and nonmilk oral intake estimates, median curves for each site were estimated using generalized additive models for location, scale, and shape and the *GAMLSS* package (V5.4-20) using age (in d) as the only explanatory variable and with penalized β-splines. Full details of model estimates are provided in the Introductory paper to this series [20]. Plots are presented with study sites highlighted separately. Associations between milk volumes and concentrations of milk nutrients were estimated by weighted rank correlations.

## Results

**FIGURE 1:**
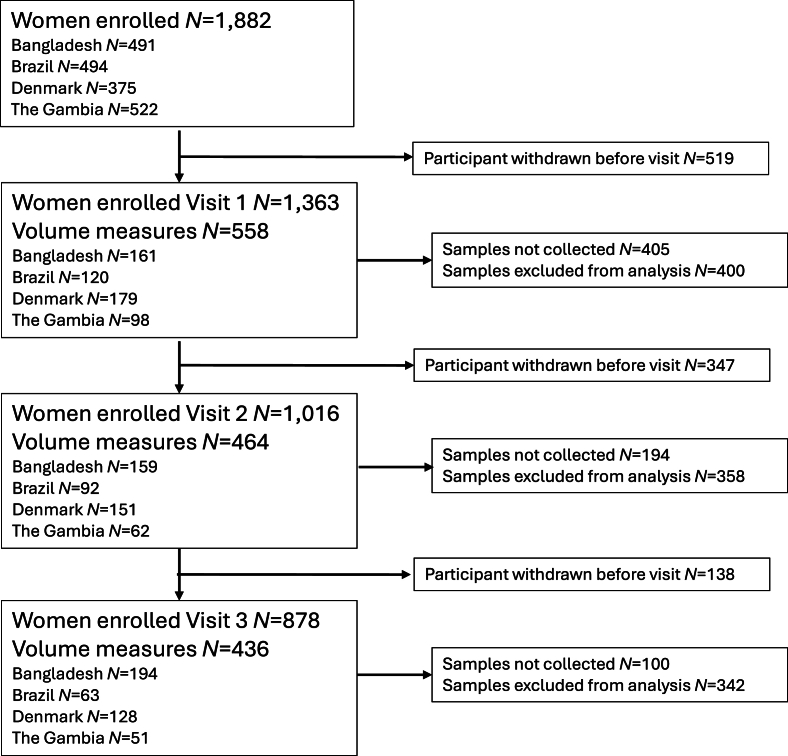
Participant flow chart for human milk volume assessment by study time point and site.

A total of 1458 data points contributed to the milk volume analysis for the MILQ study. The number of mother–infant dyads contributing data decreased between study visits due to participant exclusions [21], missed milk intake assessments, or data exclusion due to invalid volume estimates (Figure 1). Baseline characteristics (visit M1) of women included in the milk volume analyses are described in Table 1.

**TABLE 1:** Participant characteristics for women with milk volume data available at M1.

		All sites (*N* = 558)		Bangladesh (*N* = 161)		Brazil (*N* = 120)		Denmark (*N* = 179)		The Gambia (*N* = 98)	
		Mean (SD)	*N* (%)	Mean (SD)	*N* (%)	Mean (SD)	*N* (%)	Mean (SD)	*N* (%)	Mean (SD)	*N* (%)
Age (y)		27.9 (5.4)		23.8 (4.0)		27.6 (6.0)		31.4 (3.3)		28.8 (5.1)	
BMI^1^ (kg/m^2^)		24.0 (3.3)		24.7 (3.4)		24.3 (3.0)		22.4 (2.4)		25.2 (3.6)	
Literacy	No		23 (4.1)		12 (7.5)		0 (0)		0 (0)		11 (11.2)
	Yes		535 (95.9)		149 (92.5)		120 (100)		179 (100)		87 (88.8)
Education	None		29 (5.2)		15 (9.3)		0 (0)		0 (0)		14 (14.3)
	Primary		77 (13.8)		55 (34.2)		16 (13.3)		0 (0)		6 (6.1)
	Secondary		259 (46.4)		88 (54.7)		90 (75.0)		9 (5.0)		72 (73.4)
	≥Graduate		193 (34.6)		3 (1.9)		14 (11.7)		170 (95.0)		6 (6.1)
Formal employment	No		321 (57.5)		157 (97.5)		64 (53.3)		23 (12.9)		77 (78.6)
	Yes		215 (38.5)		1 (0.6)		53 (44.2)		144 (80.5)		17 (17.4)
	Student		22 (3.9)		3 (1.9)		3 (2.5)		12 (6.7)		4 (4.1)
Parity	0		278 (49.8)		61 (37.9)		49 (40.8)		130 (72.6)		38 (38.8)
	1		141 (25.3)		76 (47.2)		44 (36.7)		0 (0)		21 (21.4)
	2		87 (15.6)		17 (10.6)		13 (10.8)		37 (20.7)		20 (20.4)
	3		31 (5.6)		5 (3.1)		7 (5.8)		12 (6.7)		7 (7.1)
	4		9 (1.6)		1 (0.6)		1 (0.8)		0 (0)		7 (7.1)
	5+		12 (2.2)		1 (0.6)		6 (5.0)		0 (0)		5 (5.1)

1 BMI, body mass index, calculated using pregestational BMI for Brazil and Denmark; BMI at screening based on weight and height for Bangladesh and The Gambia.

Table 2 shows intakes of human milk by country and sample collection time point (1–8.5 mo). Mean human milk intake for all samples pooled (time points and country) was 781 g/d. For all time points pooled, human milk intakes were highest in Brazil and lowest in Bangladesh, but patterns were variable between sites when time points were looked at individually. Statistical comparisons of intakes across time points of collection (e.g., months 1.0–3.49 compared with months 6.0–8.5) have not been made due to the inclusion criteria differing by study visit concerning infant feeding practice.

**TABLE 2:** Estimated human milk intakes by country and time point.

	All sites^1^	Bangladesh	Brazil	Denmark	The Gambia
	Milk intake (g/d)				
M1 (1–3.49 mo)	*N* = 558^2^ 818 (152)	*N* = 161 815 (152)	*N* = 120 843 (155)	*N* = 179 811 (148)	*N* = 98 804 (149)
M2 (3.5–5.99 mo)	*N* = 464 833 (186)	*N* = 159 775 (186)	*N* = 92 856 (199)	*N =* 151 852 (172)	*N* = 62 850 (175)
M3 (6–8.5 mo)	*N* = 436 691 (206)	*N* = 194 618 (181)	*N* = 63 740 (203)	*N =* 128 652 (216)	*N* = 51 755 (189)
Pooled (1–8.5 mo)	*N* = 1458 781 (193)	*N* = 514 736 (193)	*N* = 275 813 (194)	*N =* 458 771 (200)	*N* = 211 803 (179)

1 Data for Bangladesh, Brazil, and The Gambia were generated by the stable isotope dilution dose-to-mother method, which enables estimation of both human milk and nonmilk oral intakes. In Denmark, 24-h test weighing was used, so only human milk intake was measured.

2 Data are sample number (*N*), means, and (SD).

**FIGURE 2:**
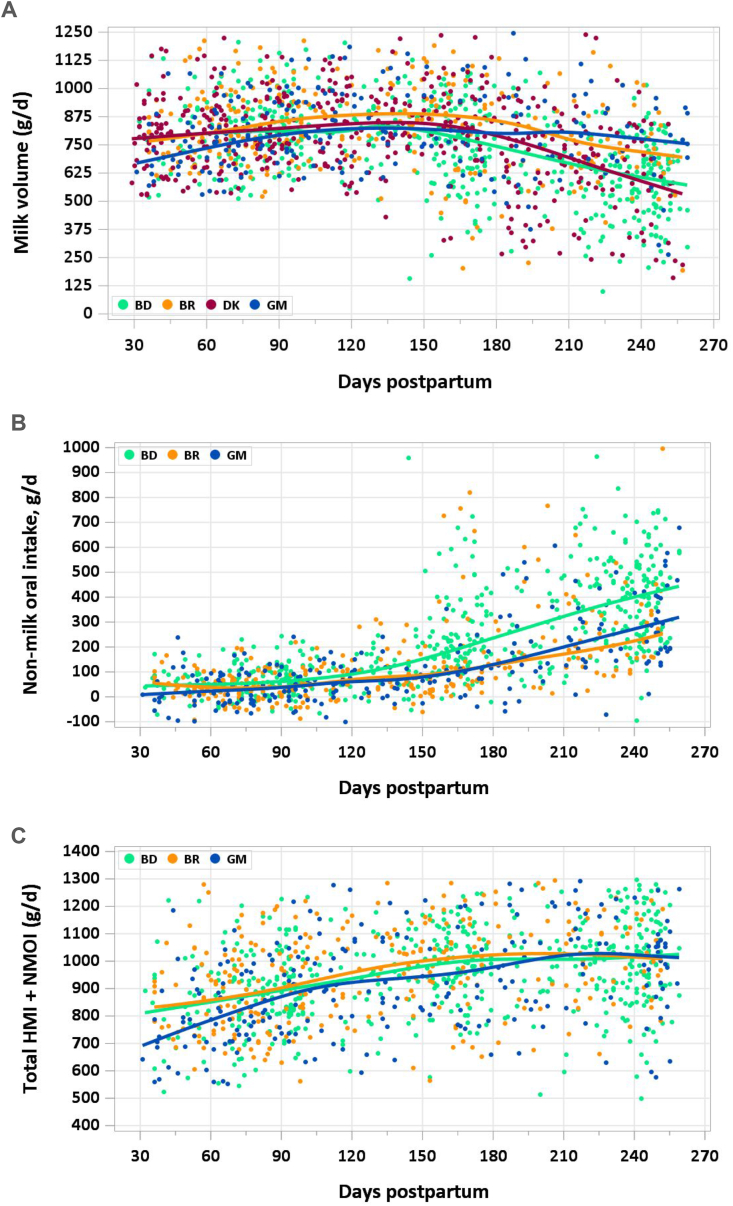
Human milk (A), nonmilk (B), and combined milk and nonmilk (C) intake from 1-8.5 mo postpartum. In Denmark, nonmilk oral intake was not measured due to use of test weighing rather than stable isotope dilution dose-to-mother method. BD, Bangladesh; BR, Brazil; DK, Denmark; GM, The Gambia.

Figure 2 shows human milk (Figure 2A), NMOI (Figure 2B), and human milk+NMOI (Figure 2C) plotted by time with countries separated by color. Milk intakes remain somewhat stable across the first 8.5 mo of lactation, with evidence of a decrease from ∼7 mo postpartum. This contrasts with a gradual increase in NMOI and combined intake of human milk and NMOI (**2c**). From 1–3.49 mo, intakes across sites were highly consistent. From 3.5 mo, intakes in Bangladesh were lower than in the other 3 countries; by 6 mo, both Bangladesh and Denmark had lower intakes than Brazil and The Gambia.

Table 3 presents data from visits 2 and 3 according to the reported mode of feeding (exclusive compared with mixed feeding). Visit 1 data are not included in this Table as all women reported exclusively breastfeeding at this time. Mean intakes among women reporting exclusive breastfeeding across all time points were 820 g/d compared with 680 g/d among women who reported nonexclusively breastfeeding (*P* < 0.01).

**TABLE 3:** Intakes of human milk by reported feeding status.

Site	M1 (1–3.49 mo)	M2 (3.5–5.99 mo)		M3 (6–8.5 mo)	
	EBF^3^ [556]^4^	EBF [278]	Mixed-fed [185]	EBF [15]	Mixed-fed [420]
All^1^	818 (152) [556]	870 (158)	773 (212)	777 (147)	687 (207)
Bangladesh	815 (152) [161]	846 (143) [91]	681 (195) [68]	730 (105) [4]	615 (181) [190]
Brazil	841 (156) [118]	919 (150) [45]	797 (220) [47]	855 (206) [4]	732 (200) [59]
Denmark^2^	811 (148) [179]	874 (158) [88]	820 (184) [63]	780 (na) [1]	653 (215) [126]
The Gambia	804 (149) [98]	855 (166) [54]	845 (224) [7]	743 (95) [6]	756 (198) [45]

1 Pooled means are weighted.

2 Data for Denmark are corrected by a factor of 1.05 to account for insensible water losses during the period of test weighing. All other sites used the stable isotope dilution dose-to-mother method.

3 Exclusively breastfed.

4 Values reported as means and (SD) and [*N*].

**FIGURE 3:**
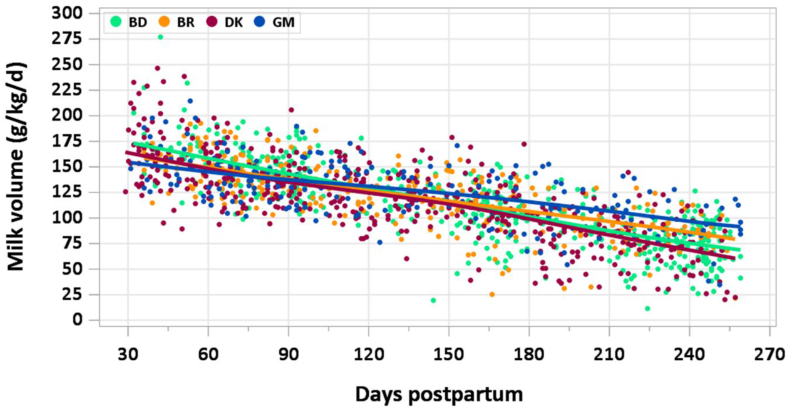
Human milk intake per body weight (g/kg per day) across months 1 to 8.5 of lactation. In Denmark, nonmilk oral intake was not measured due to use of test weighing rather than the stable isotope dilution dose-to-mother method. BD, Bangladesh; BR, Brazil; DK, Denmark; GM, The Gambia.

Mean (±SD) infant weight increased from 5.58 kg (±0.84) at 1 to 3.49 mo to 7.33 (±0.90) kg at 3.5 to 5.99 mo, and to 8.18 (0.98) kg at 6 to 8.5 mo. When human milk intake is adjusted for infant weight, a marked decrease is observed across the period of lactation under observation (Figure 3). Mean intake per kg infant body weight decreased from 147 (±25.4) g/kg/d at 1 to 3.49 mo to 118 (±25.0) g/kg/d at 3.5 to 5.99 mo to 86 (±25.4) kg at 6 to 8.5 mo. Little variation was observed among countries.

Table 4 presents correlations of milk intakes against human milk nutrient concentrations by study visit. In the first column, the weighted rank correlations between milk volume and individual nutrient concentrations are detailed, and in the second column, milk volume is correlated against the total daily nutrient intake (i.e., the amount of each nutrient the infant would receive based on milk intake estimates).

**TABLE 4:** Correlation between human milk volume and milk nutrient concentrations.

Milk nutrient	Correlation^1^ of milk volume with nutrient concentration			Correlation of milk volume with total daily intake of nutrient		
	1–3.49 mo	3.5–5.99 mo	6–8.5 mo	1–3.49 mo	3.5–5.99 mo	6–8.5 mo
Protein (g/L)	−0.24	−0.13	−0.18	0.47	0.54	0.69
Carbohydrate (g/L)	−0.02	0.05	0.07	0.95	0.93	0.97
Fat (g/L)	−0.10	−0.16	−0.13	0.32	0.27	0.46
Energy (kcal/L)	−0.12	−0.16	−0.13	0.62	0.59	0.74
Vitamin B1 (ug/L)	0.00	0.03	0.14	0.48	0.52	0.68
Vitamin B2 (ug/L)	−0.07	−0.15	−0.07	0.34	0.29	0.48
Vitamin B3 (mg/L)	−0.17	−0.19	−0.14	0.26	0.25	0.45
Pantothenic acid (mg/L)	−0.14	−0.14	−0.07	0.38	0.40	0.51
Vitamin B6 (ug/L)	0.09	0.05	0.05	0.40	0.45	0.50
Biotin (ug/L)	−0.06	−0.05	−0.05	0.25	0.31	0.35
Total choline (mg/L)	−0.03	0.07	0.12	0.47	0.56	0.63
Vitamin B12 (pmol/L)	−0.13	−0.21	−0.18	0.25	0.23	0.36
Sodium (mg/L)	−0.15	−0.12	−0.20	0.29	0.37	0.42
Magnesium (mg/L)	−0.05	−0.06	−0.02	0.63	0.69	0.78
Phosphorus (mg/L)	−0.01	−0.05	0.07	0.70	0.70	0.82
Potassium (mg/L)	−0.11	−0.13	−0.13	0.68	0.71	0.82
Calcium (mg/L)	0.03	0.06	0.16	0.70	0.74	0.83
Chromium (ug/L)	0.01	−0.01	−0.02	0.42	0.47	0.60
Iron (mg/L)	−0.18	−0.16	−0.18	0.24	0.29	0.41
Copper (mg/L)	−0.15	−0.05	0.06	0.40	0.46	0.57
Zinc (mg/L)	−0.08	0.05	0.20	0.29	0.40	0.59
Selenium (ug/L)	−0.23	−0.19	−0.17	0.40	0.48	0.65
Iodine (ug/L)	−0.07	−0.02	−0.07	0.33	0.41	0.47
Vitamin A (retinol) (umol/L)	−0.10	−0.16	−0.22	0.23	0.26	0.29
ɣ-tocopherol (mg/L)	−0.04	−0.06	−0.14	0.25	0.30	0.37
ɑ-tocopherol (mg/L)	−0.03	−0.18	−0.22	0.27	0.19	0.25
Anti-rachitic activity (IU/L)	−0.06	−0.19	−0.17	0.25	0.19	0.38

1 Data are weighted rank correlations controlled for individual differences between-site means of milk volume and the nutrient variables. Absolute correlations >0.16, 0.18, or 0.20 are significant at *P* = 0.05, 0.01, or 0.001, respectively, following Bonferroni adjustment for the 150 tests.

When milk volumes were compared against nutrient concentrations, weak and mostly negative correlations were observed across all nutrients, illustrating that milk concentrations do not change according to daily milk intakes. When volumes were compared against the total daily intake of the nutrient (volume × concentration), a much stronger, positive correlation resulted across nutrients, indicating that infants with greater milk intakes receive more nutrients each day. Scatterplots of nutrient concentrations against milk volume (Supplemental Figures 1A–27A) and total daily nutrient intake against milk volume (Supplemental Figures 1B–27B) are provided as Supplementary Materials.

## Discussion

We report intakes of human milk from 1 to 8.5 mo lactation from 4 study sites. In Bangladesh, Brazil, and The Gambia, intakes were determined using the stable isotope dilution “dose-to-mother” method, and in Denmark, before- and after-feed test weighing was used. Our data are highly consistent with the published literature, showing that intakes vary between-individual infants but with mean intakes of ∼780 g/d, matching previous global estimates. However, as previous estimates have used pooled data from multiple time points across lactation, the repeated sampling protocol implemented in the MILQ study, together with detailed data on feeding practices, allows a greater precision to our understanding of how human milk intakes change across lactation and with the introduction of nonhuman milk feeds. A novel and perhaps surprising finding from the analyses presented here is that milk nutrient concentrations consistently show little correlation with daily milk volumes. Thus, infants who habitually consume greater volumes of milk receive higher intakes of nutrients.

As highlighted, the test weighing method is believed to slightly underestimate milk intake, thus a 5% correction factor was applied to the Danish milk intake data. With this applied, intakes in early lactation — when exclusive breastfeeding was practiced — were highly consistent across the 4 site contexts, supporting the value of employing the test weighing method in contexts where the stable isotope dilution dose-to-mother technique is not feasible (due to cost, participant burden, access to analytical facilities, etc.). Further, test weighing saw fewer data losses due to logistical or analytical issues.

A benefit of the stable isotope dilution dose-to-mother method is that, in addition to estimating milk intakes, it also provides an estimate of nonmilk oral intake and therefore allows a consideration of the introduction of nonmilk feeds as women transition from exclusive breastfeeding. Using the data obtained, we observed that, after the initial study visit (where inclusion was based on EBF), milk intakes declined, whereas total intakes (milk plus nonmilk intakes) increased, reflecting a greater contribution from nonmilk intakes. From 1 to 3.49 mo, intakes did not vary by site. However, by 6 to 8.5 mo, Brazil and The Gambia had comparatively higher intakes of human milk, suggesting a greater contribution to total intakes.

The primary objective of the MILQ study was to develop RVs for human milk nutrients. Prior to MILQ, little was known about the relationship between variations in milk intake and milk concentrations, e.g., whether concentrations in milk are regulated in response to the volume of milk being habitually consumed by young infants. A previous study conducted among 212 lactating women from Indonesia looked at the relationship between milk volume and a limited set of micronutrients at 2 and 5 mo postpartum, with similar findings [22]. This study has shown that there is little evidence that the concentration of human milk nutrients varies by the volume consumed. Thus, infants who regularly consume larger volumes of milk will have intakes above those consuming smaller volumes. The implications of this for infant status need consideration, although it might be assumed that within a normal range of milk concentrations, infants will receive sufficient nutrient intakes. Infants most at risk are those consuming lower volumes and from mothers who themselves are deficient in key nutrients during lactation. This finding also reiterates the importance of assessing milk intake in studies designed to evaluate the impact of interventions on milk nutrient concentrations and infant status.

In relation to the data presented in this manuscript, the MILQ study has several strengths, including the longitudinal collection of milk intake data across 4 population groups, using aligned methods in 3 sites, with comprehensive data on breast milk micronutrient concentrations. However, alongside these strengths, several limitations must be noted, including the relatively high loss of data points for milk volume due to participant attrition and incomplete sample collection/removal of suspicious data points. These losses could introduce selection bias, although the similarity between the estimated milk intake among MILQ participants and the mean value used from global estimates adds reassurance to the validity of the data obtained.

In conclusion, we present evidence that the human milk intake of the infants recruited into the MILQ study from the 4 study settings were both consistent across sites and consistent with previously published global data. Importantly, however, we present novel evidence to show that milk nutrient concentrations do not vary with the between-individual variations in milk volume.

Future planned analyses within MILQ will explore the impact this has on infant nutrient status with considerations for the recommended nutrient intakes of infants.

## Authors contributions

The authors' responsibilities were as follows - SEM, MMI, GK, KFM, LHA: designed the study, responsible for study implementation; SD, AK: responsible for oversight of stable isotope dilution, including sample analysis; JMP: analyzed data; SHC, GTS: contributed to study implementation at site level; SEM: wrote the initial draft of the paper and was responsible for final content; and all authors: read and approved the final manuscript.

## Data availability

Data described in the manuscript, code book, and analytic code will be made available upon request, pending application and approval.

## Funding

Supported by the Gates Foundation (OPP1148405/INV-002300, OPP1061055); and USDA intramural funds (2023-51530-025-00D).

## Conflict of interest

The authors report no conflicts of interest.
