# Supplementary material for: Breast Milk Intake from 1 to 8.5 Months of Lactation in the Multisite Mothers, Infants and Lactation Quality (MILQ) Study
Source: Adv Nutr. 2025 Aug 26;16(Suppl 1):100456. doi: 10.1016/j.advnut.2025.100456 (PMC12673386; doi:10.1016/j.advnut.2025.100456)
Supplement: multimedia component 1 [file mmc1.docx]

**Moore et al. Breast milk intake from 1 to 8.5 months of lactation in the multi-site Mothers, Infants and Lactation Quality (MILQ) study**

**Supplementary Figures 1 to 27**

**Scatterplots of nutrient concentrations (A) and total daily nutrient intakes (B) vs daily milk intake, for visits M1 and M2 (1-6 mo) combined, by site. BD=Bangladesh, BR=Brazil, DK=Denmark, GM=Gambia.**

**Supplementary Figure 1. Protein**

| **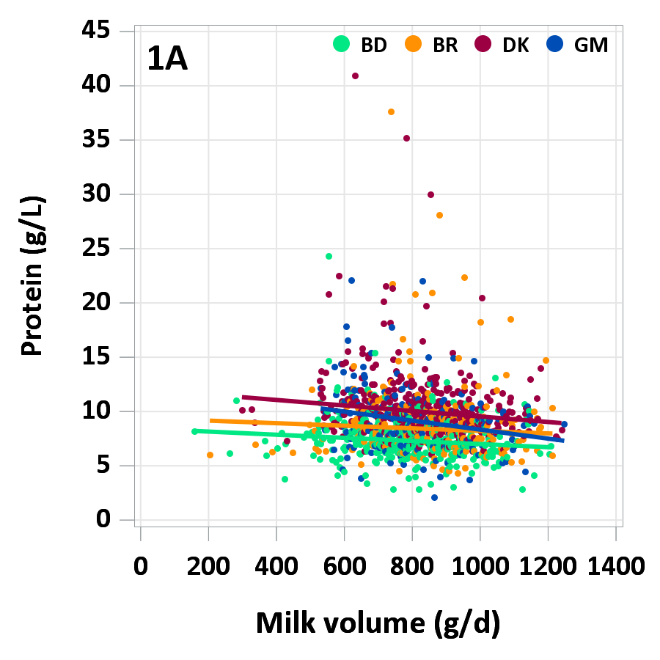** | **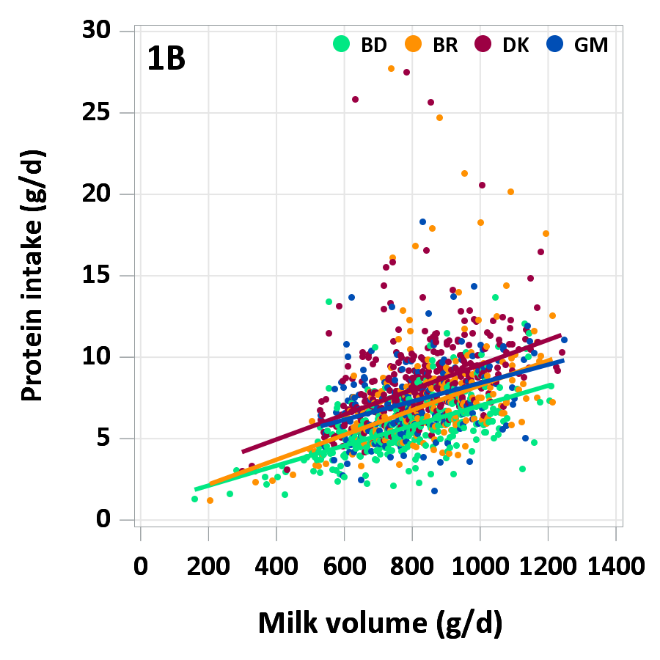** |
| --- | --- |

**Supplementary Figure 2. Carbohydrate**

| **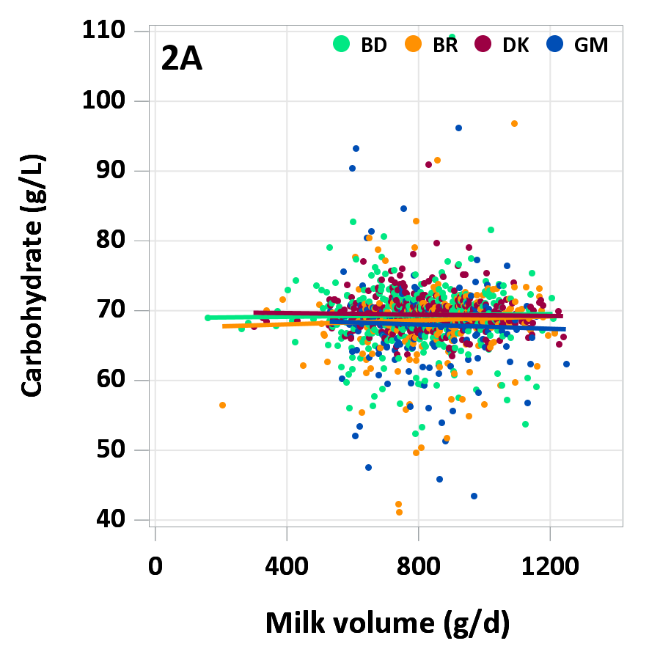** | **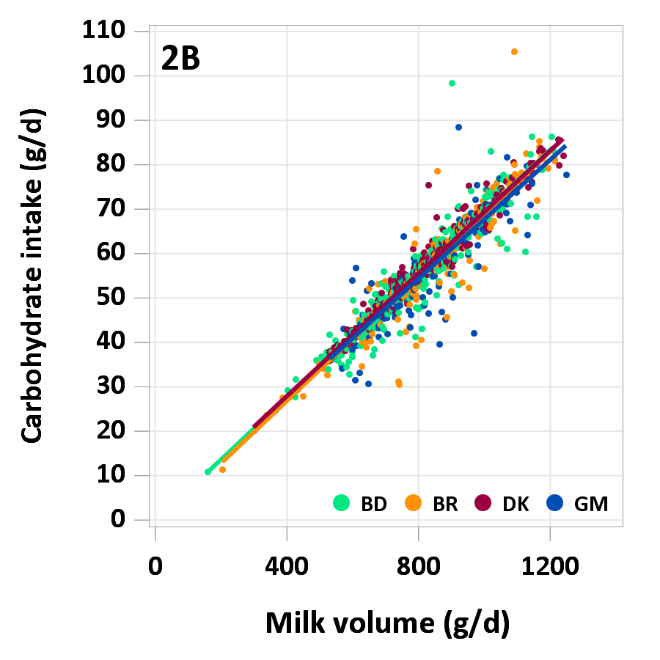** |
| --- | --- |

**Supplementary Figure 3. Fat**

| **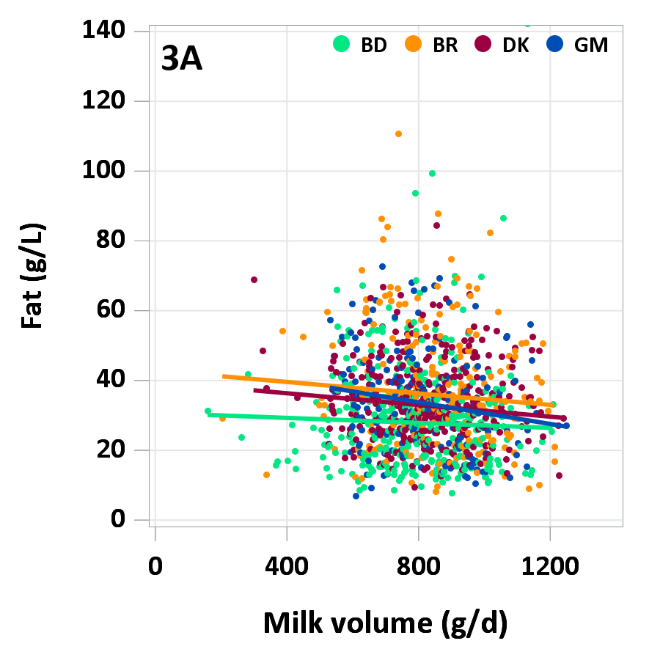** | **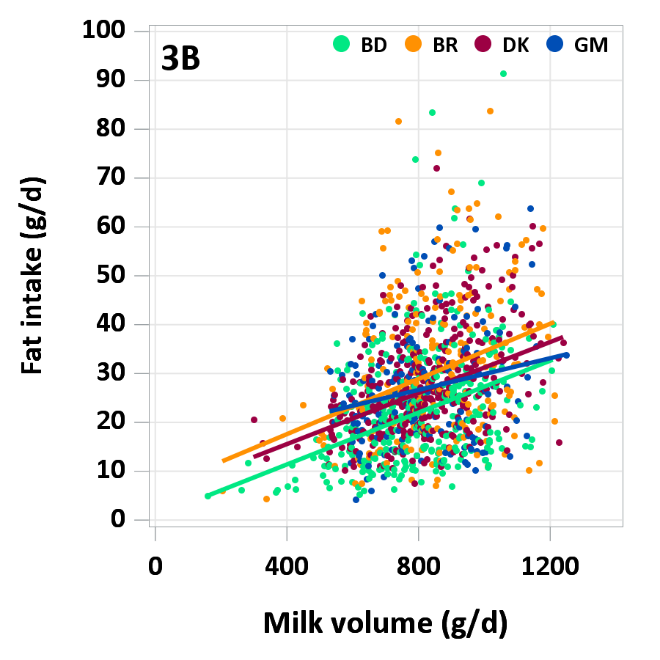** |
| --- | --- |

**Supplementary Figure 4. Energy**

| **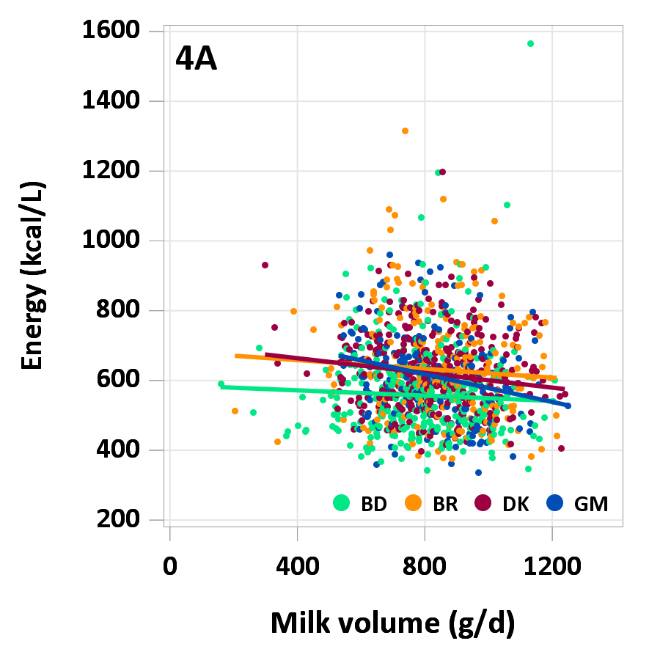** | 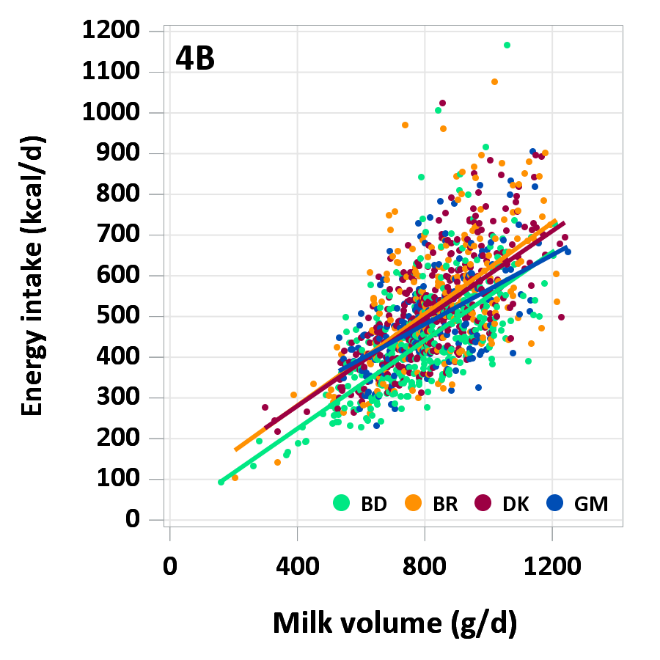 |
| --- | --- |

**Supplementary Figure 5. Vitamin B1**

| **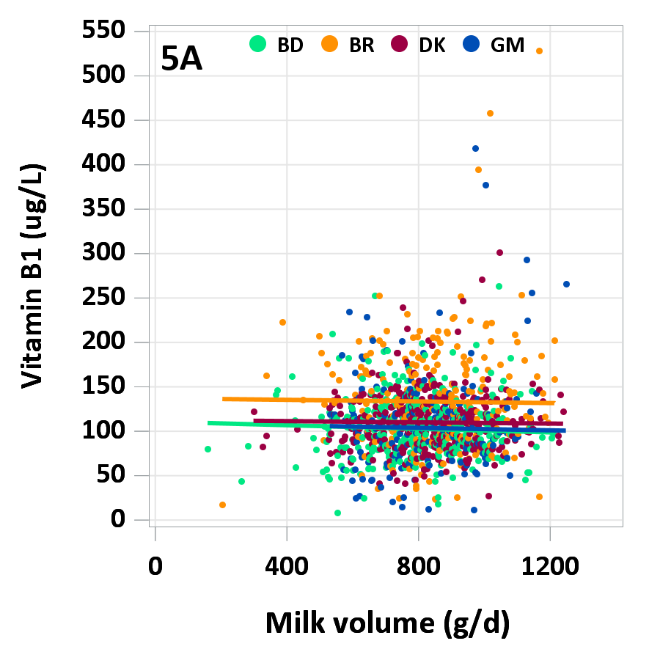** | **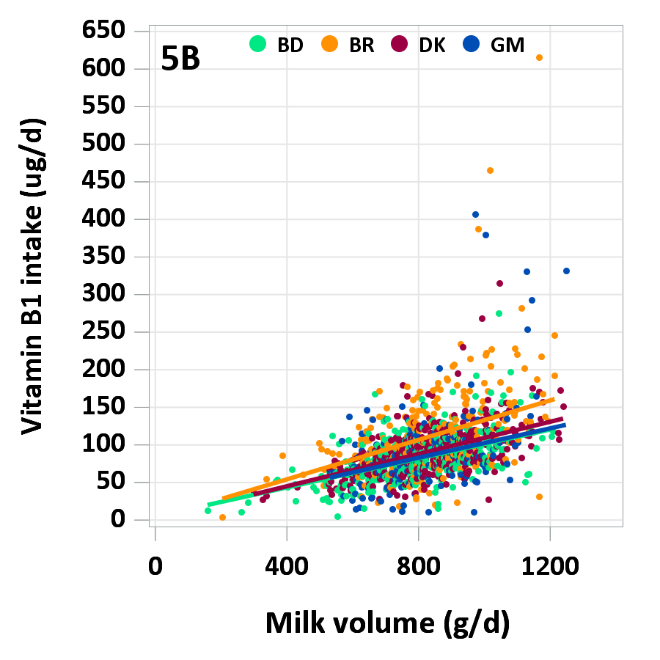** |
| --- | --- |

**Supplementary Figure 6. Vitamin B2**

| **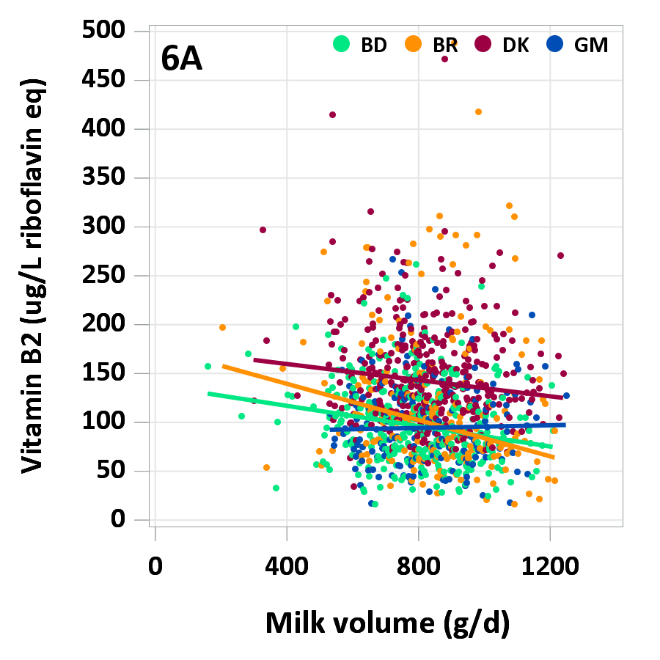** | **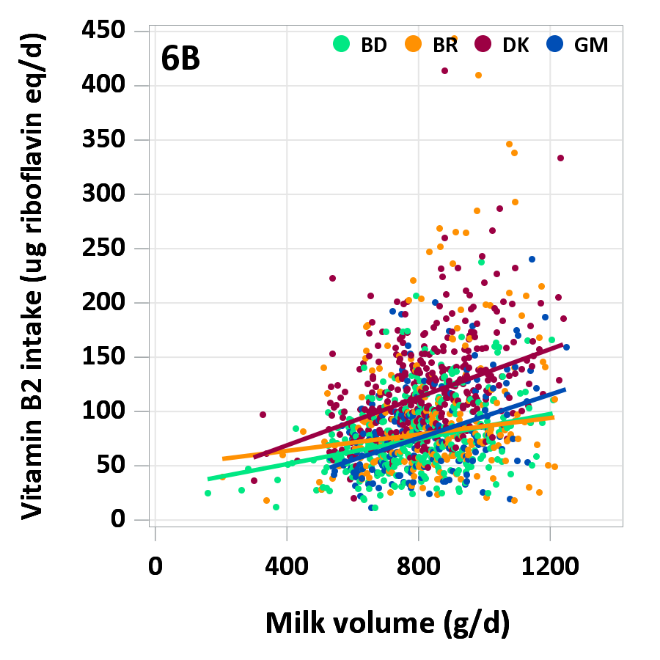** |
| --- | --- |

**Supplementary Figure 7. Vitamin B3**

| **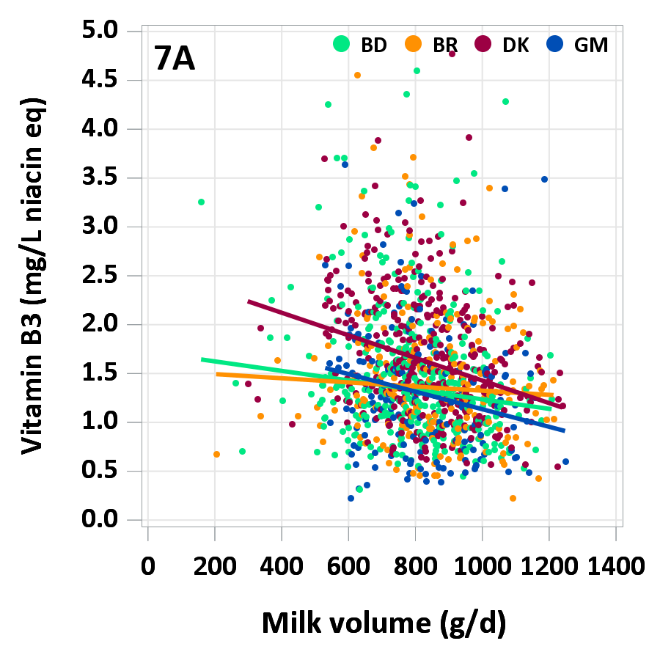** | **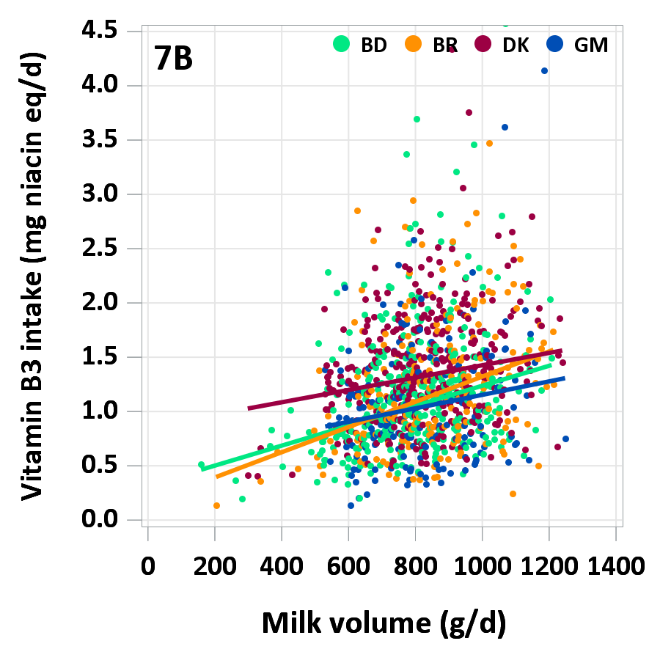** |
| --- | --- |

**Supplementary Figure 8. Pantothenic acid**

| **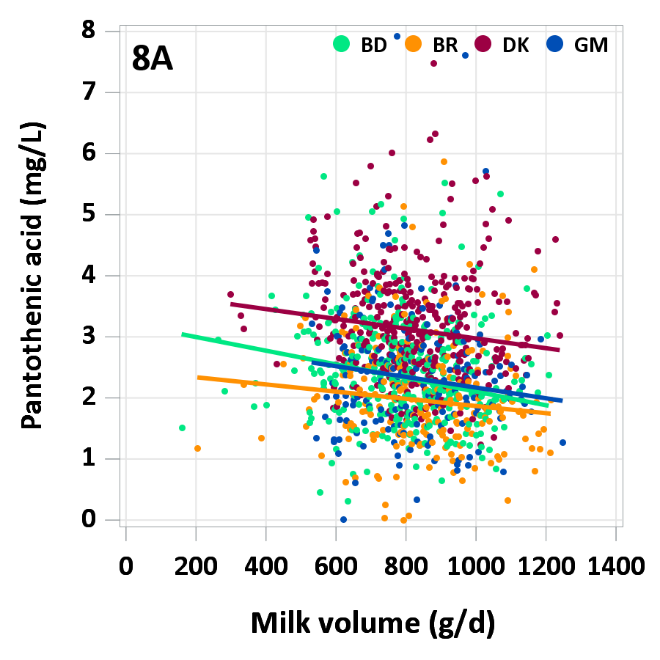** | **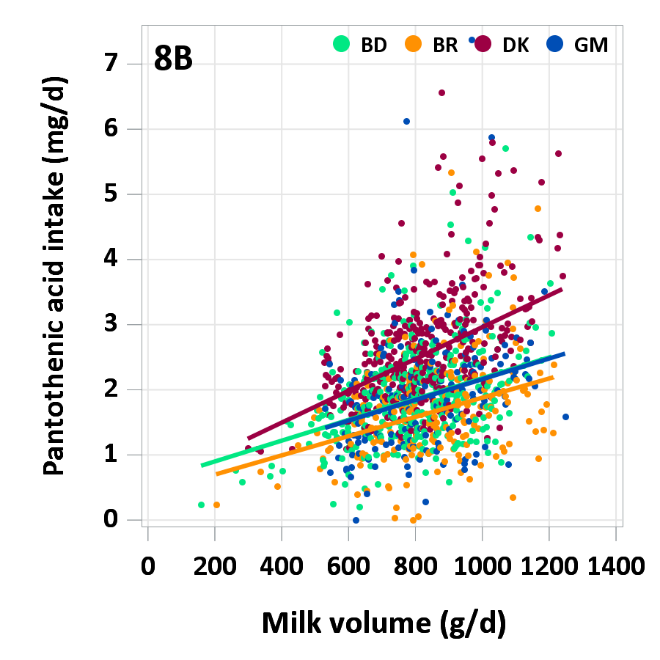** |
| --- | --- |

**Supplementary Figure 9. Vitamin B6**

| **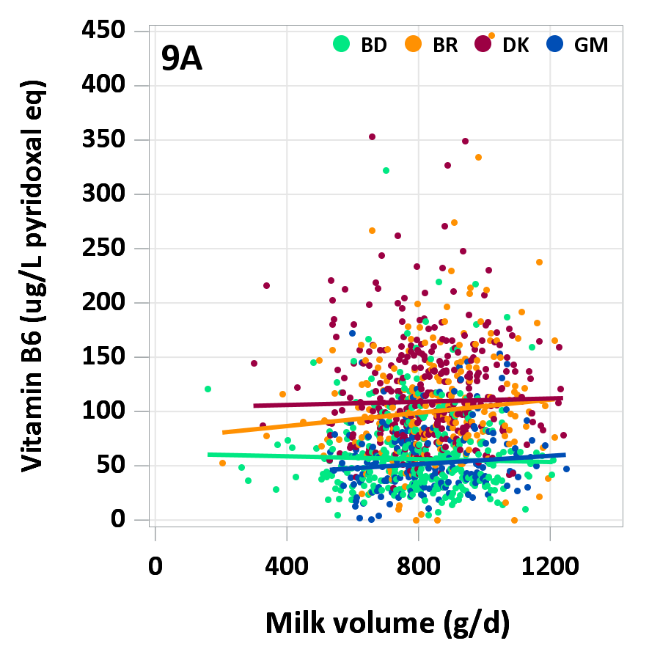** | **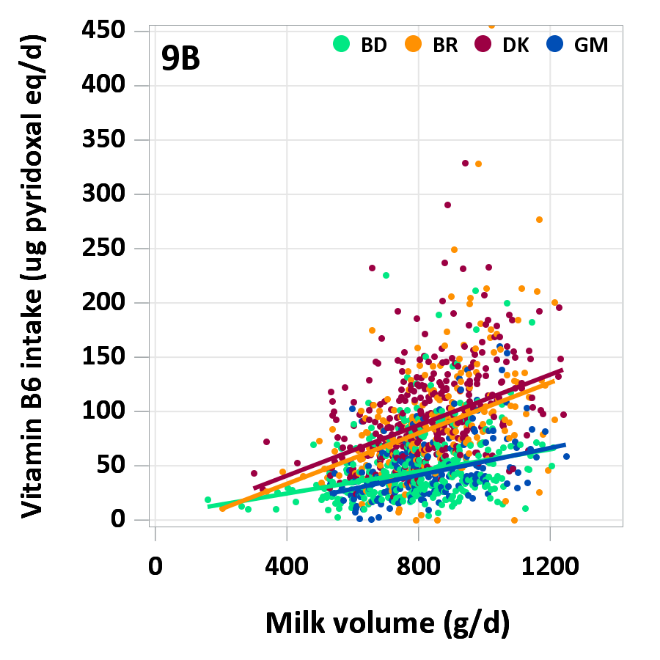** |
| --- | --- |

**Supplementary Figure 10. Biotin**

| **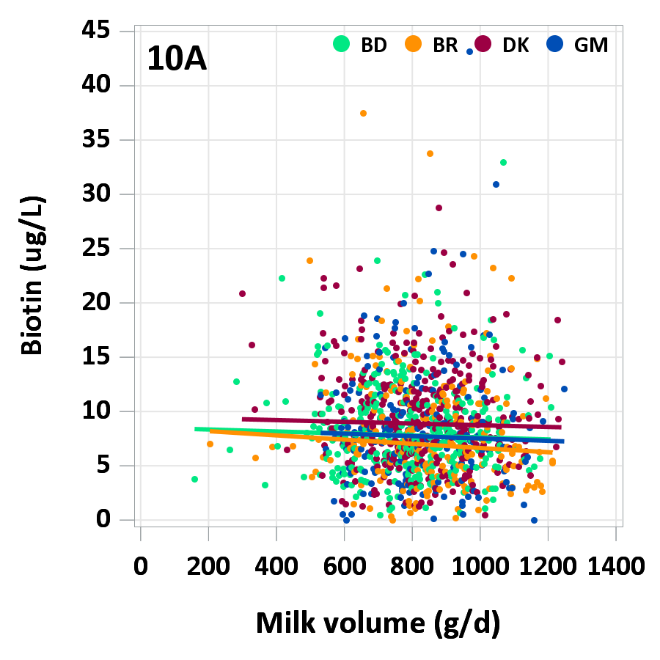** | **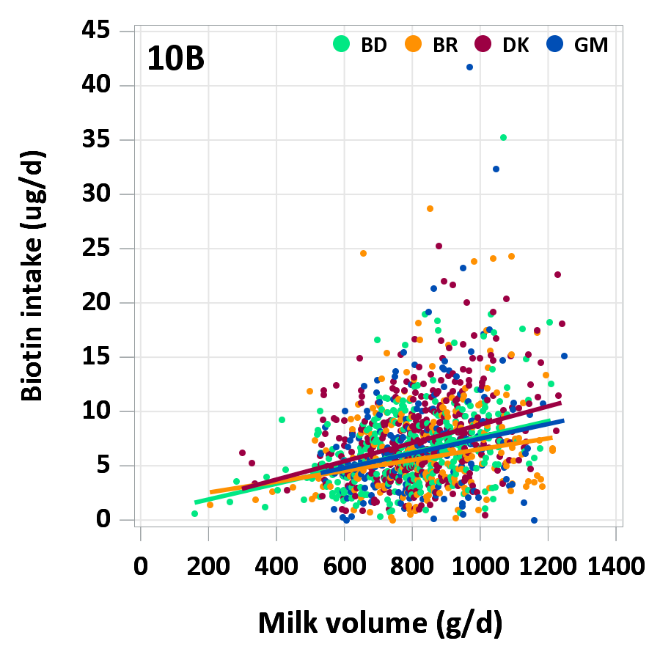** |
| --- | --- |

**Supplementary Figure 11. Total choline**

| **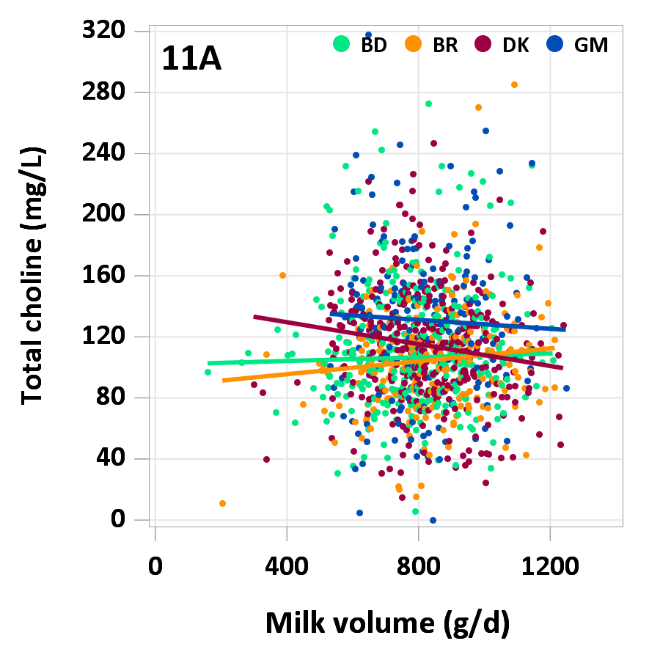** | **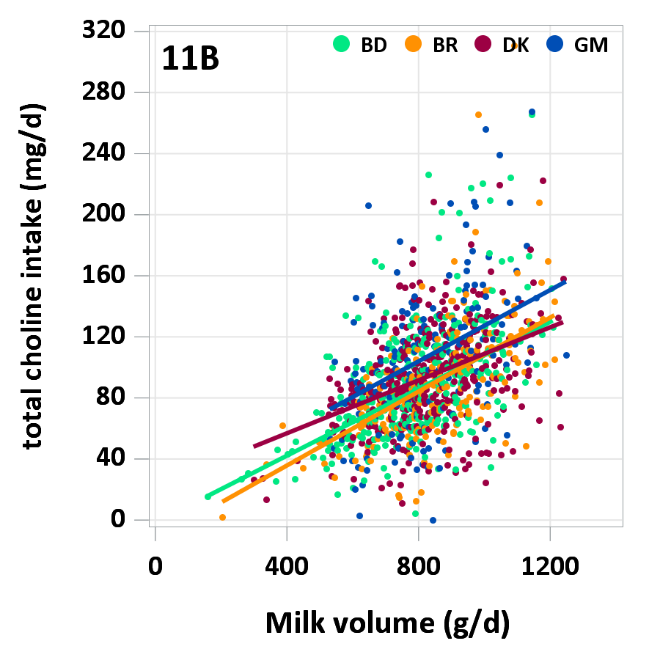** |
| --- | --- |

**Supplementary Figure 12. Vitamin B12**

| **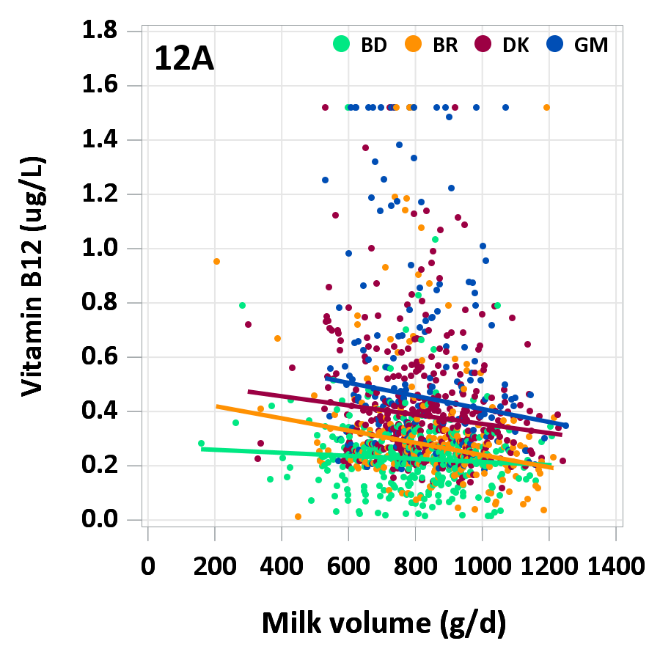** | **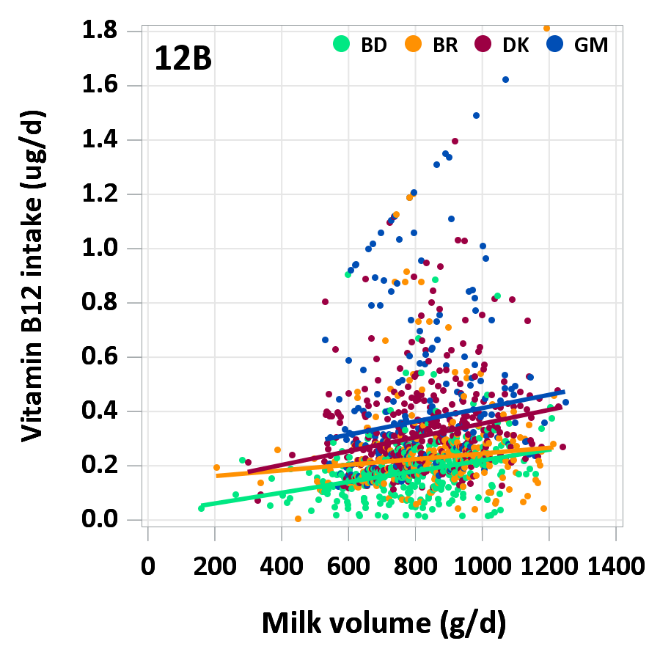** |
| --- | --- |

**Supplementary Figure 13. Sodium**

| **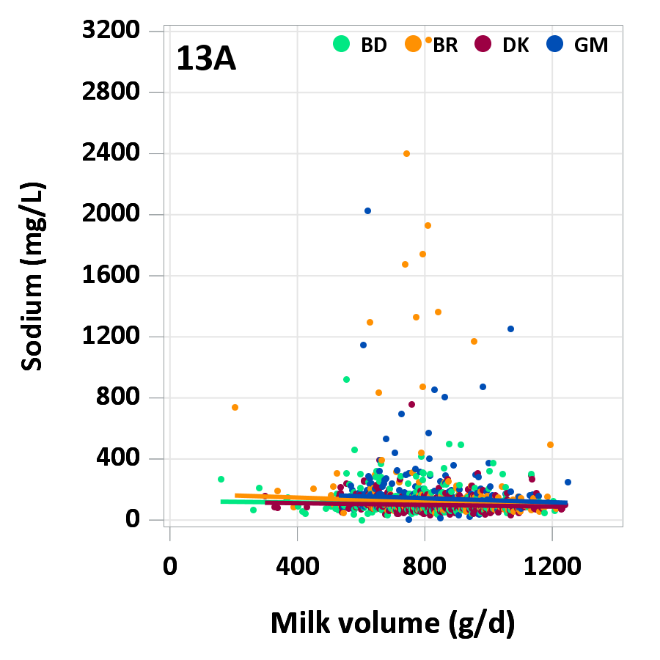** | **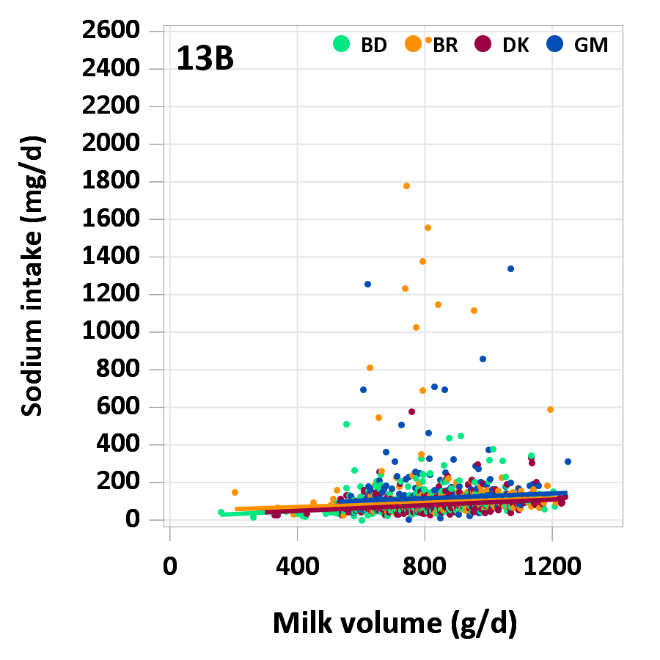** |
| --- | --- |

**Supplementary Figure 14. Magnesium**

| **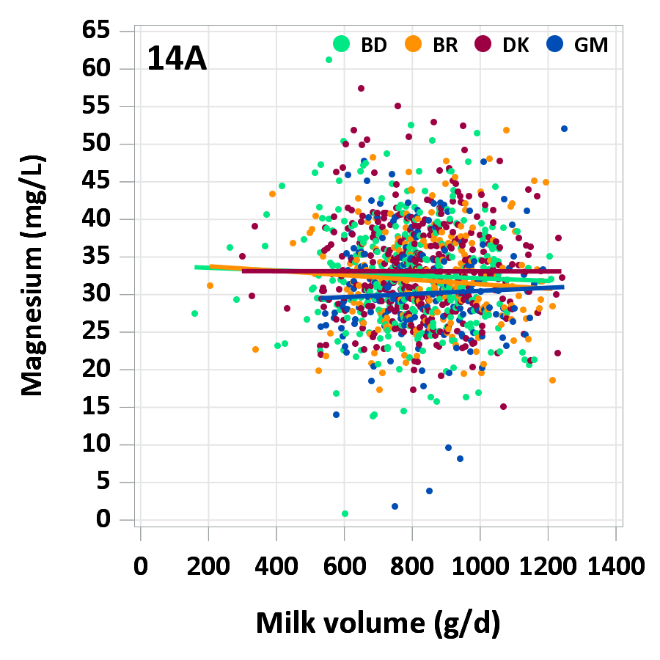** | **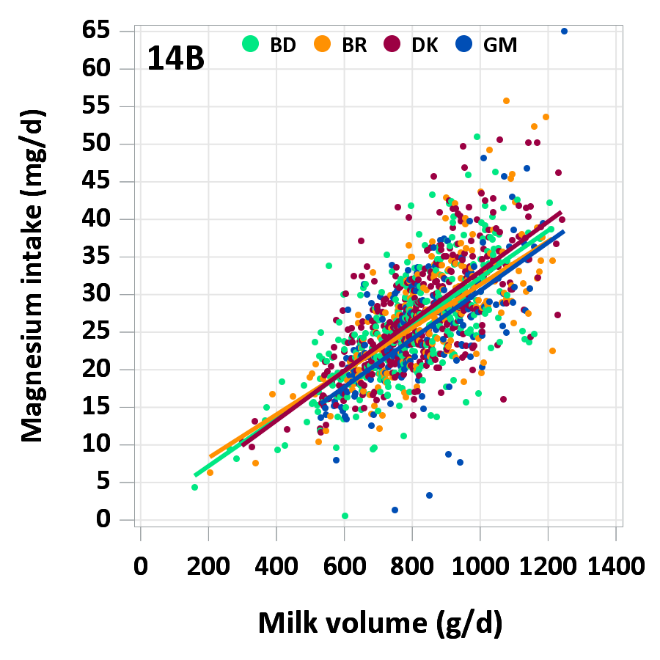** |
| --- | --- |

**Supplementary Figure 15. Phosphorus**

| **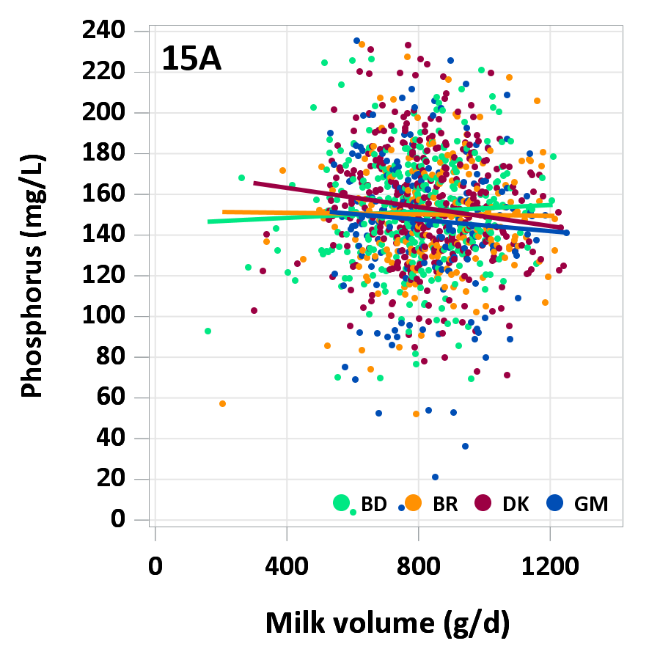** | **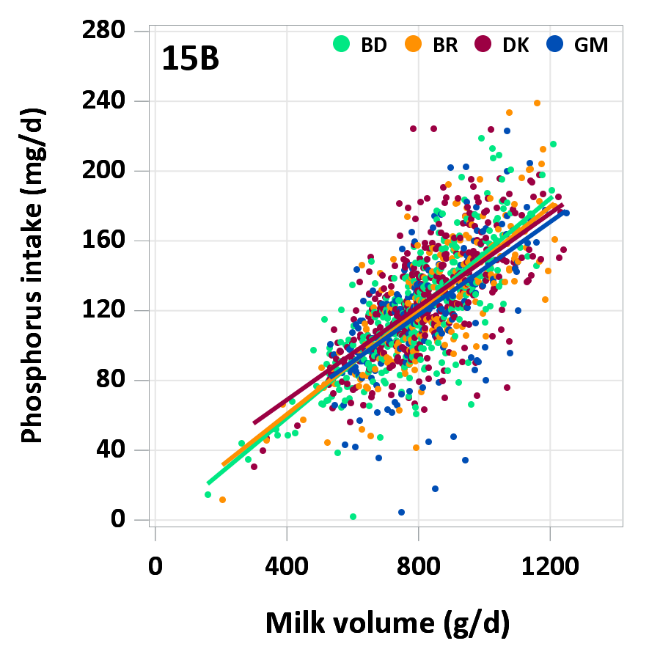** |
| --- | --- |

**Supplementary Figure 16. Potassium**

| **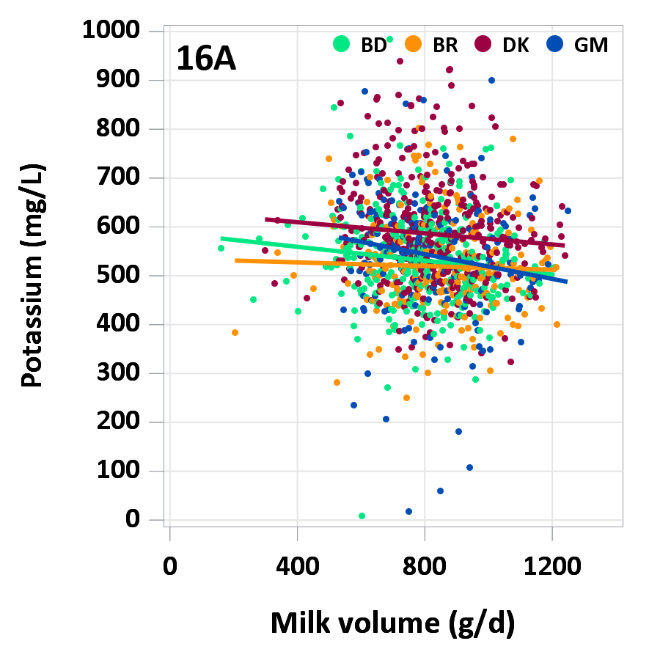** | **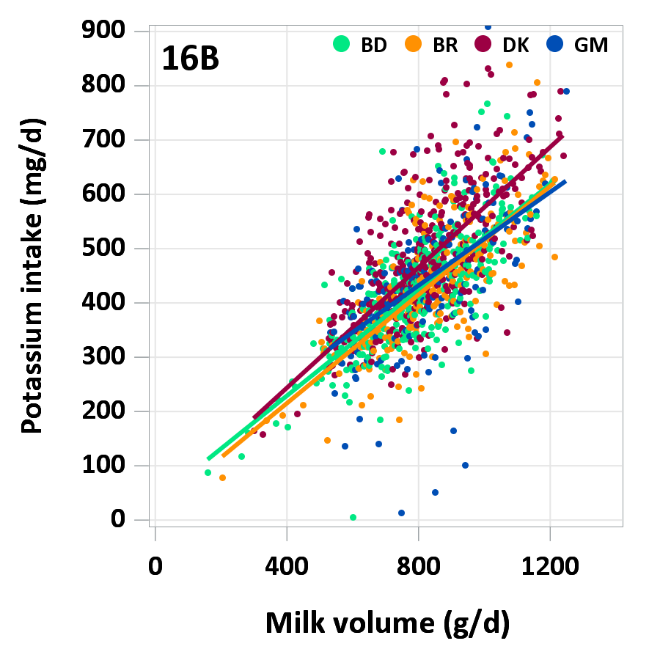** |
| --- | --- |

**Supplementary Figure 17. Calcium**

| **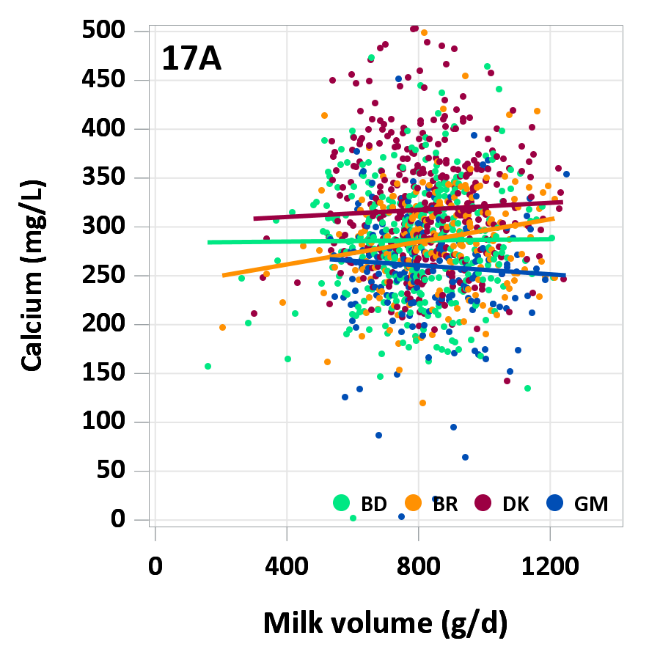** | **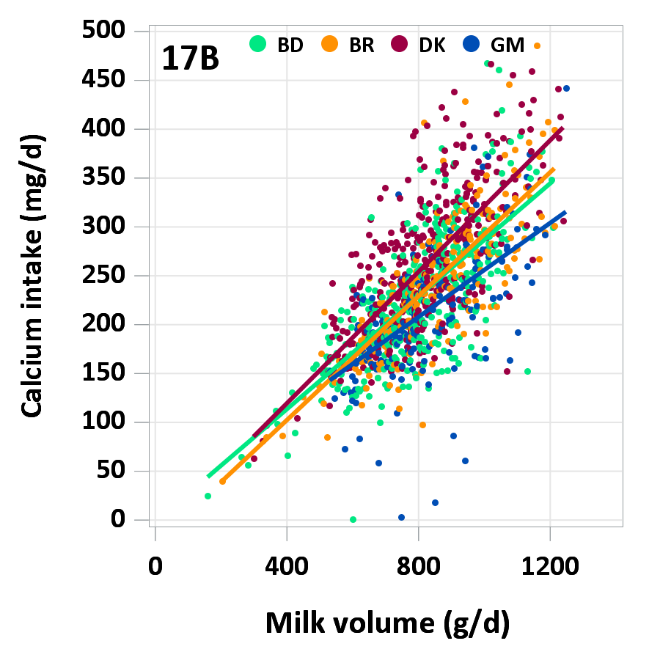** |
| --- | --- |

**Supplementary Figure 18. Chromium**

| **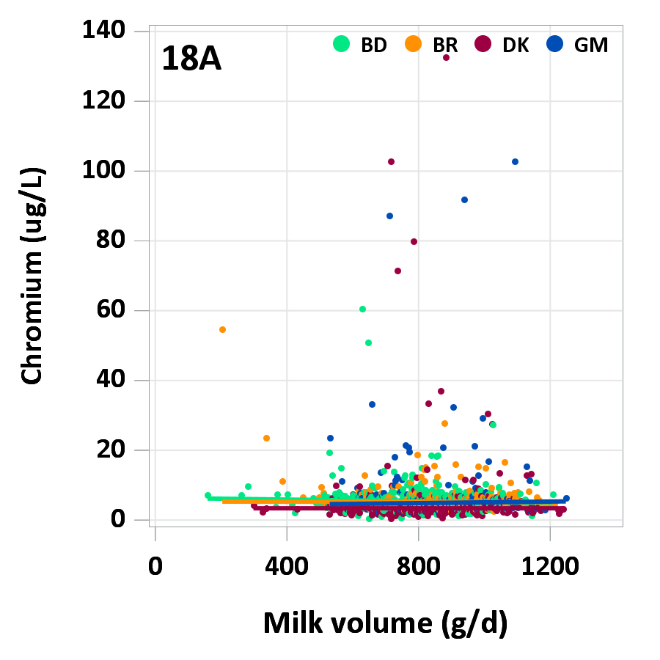** | **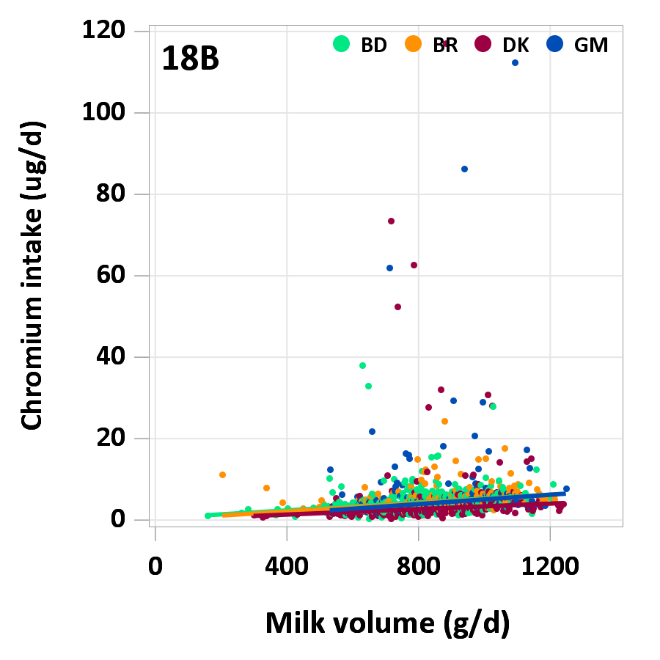** |
| --- | --- |

**Supplementary Figure 19. Iron**

| **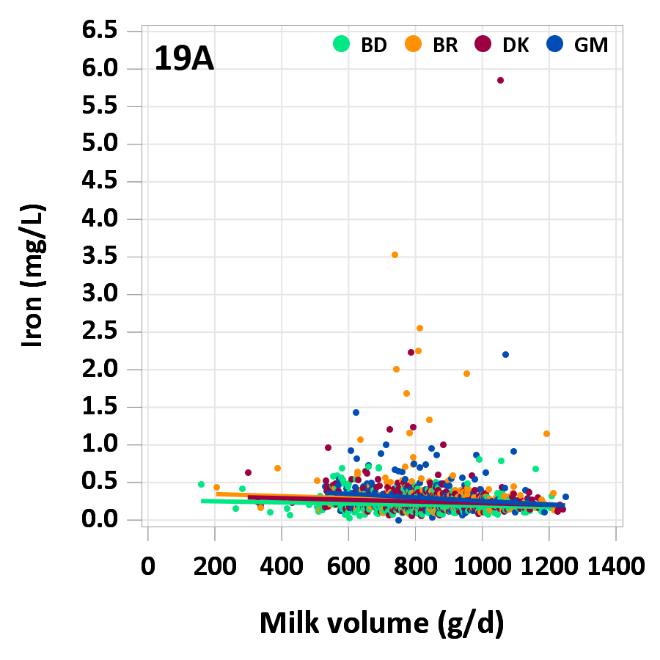** | **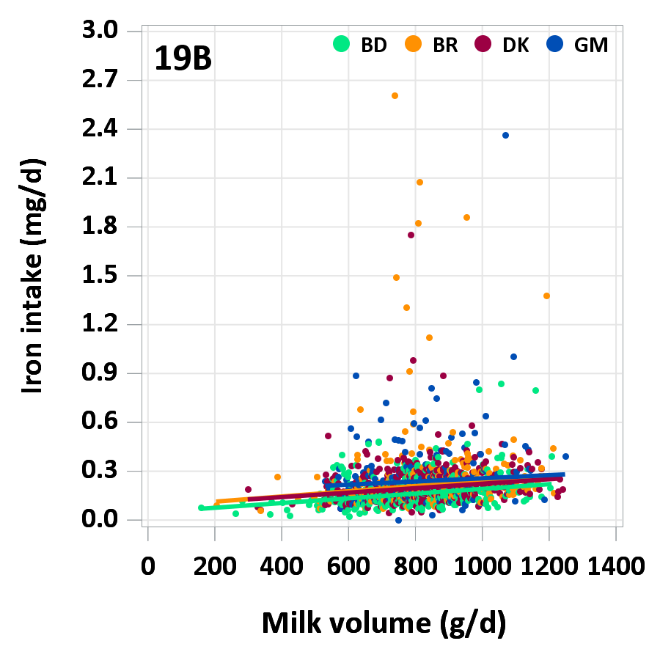** |
| --- | --- |

**Supplementary Figure 20. Copper**

| **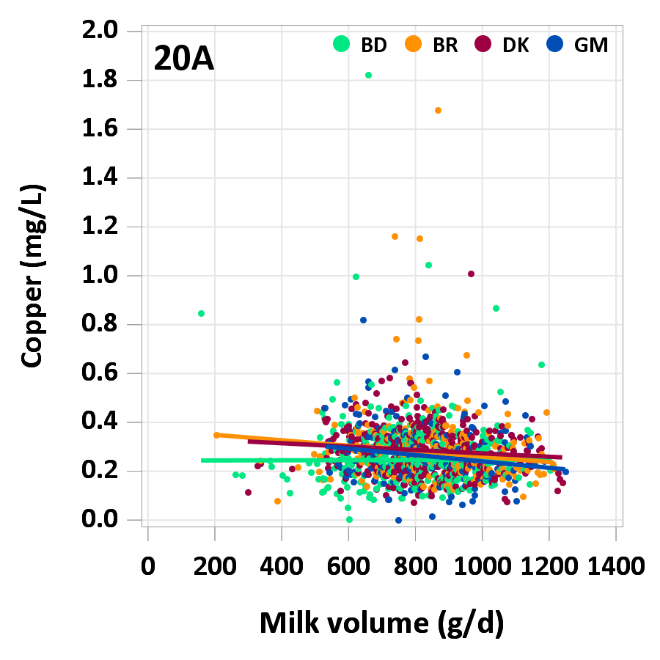** | **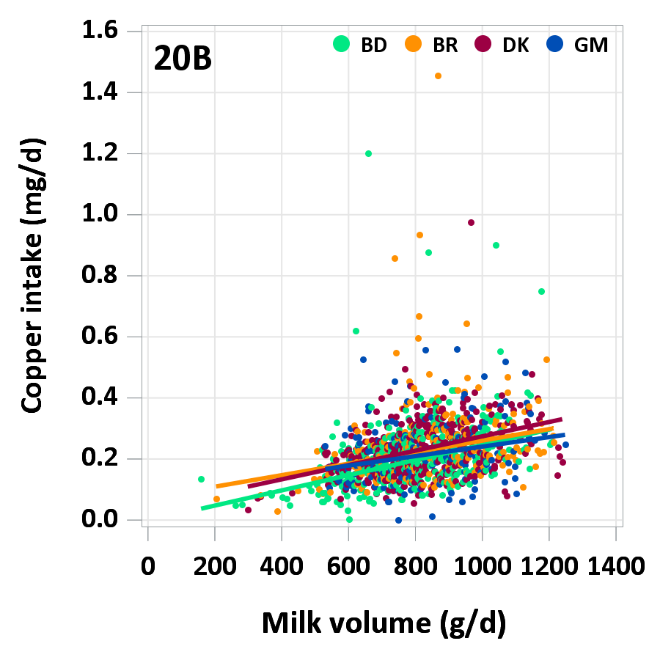** |
| --- | --- |

**Supplementary Figure 21. Zinc**

| **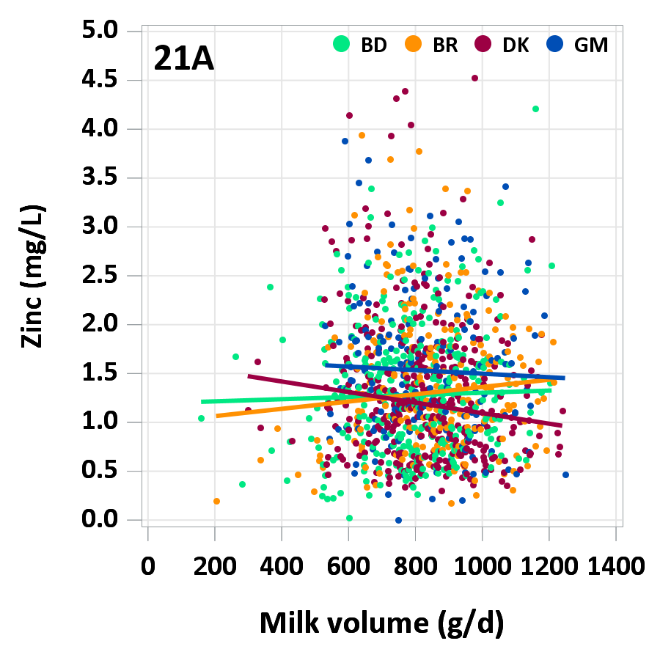** | **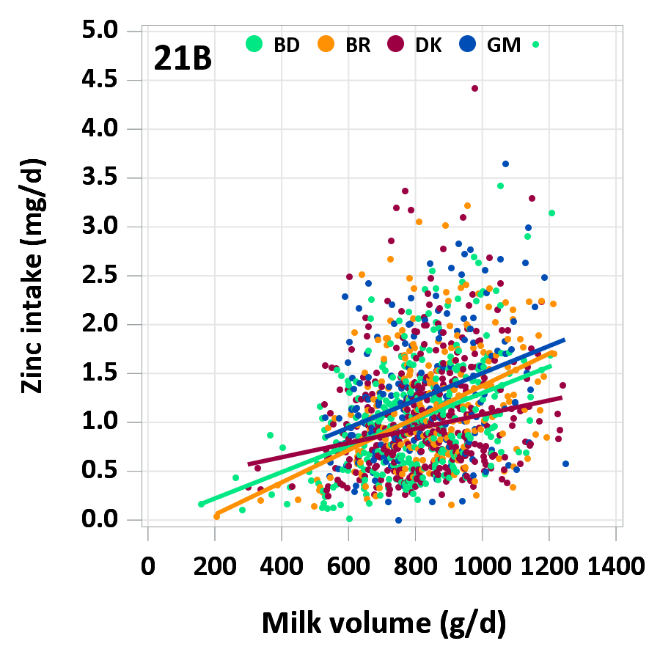** |
| --- | --- |

**Supplementary Figure 22. Selenium**

| **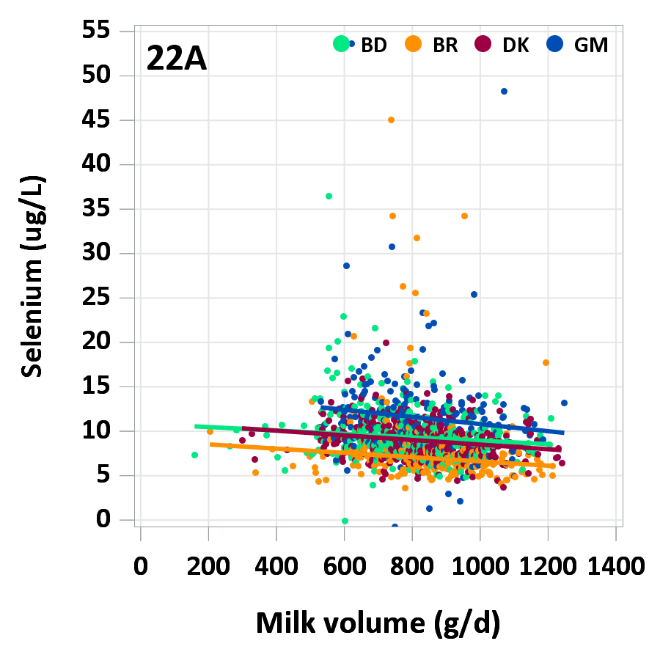** | **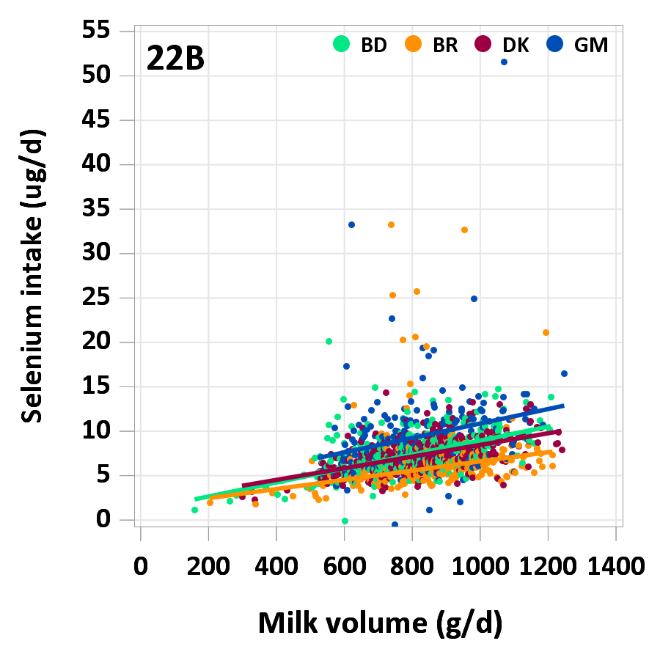** |
| --- | --- |

**Supplementary Figure 23. Iodine**

| **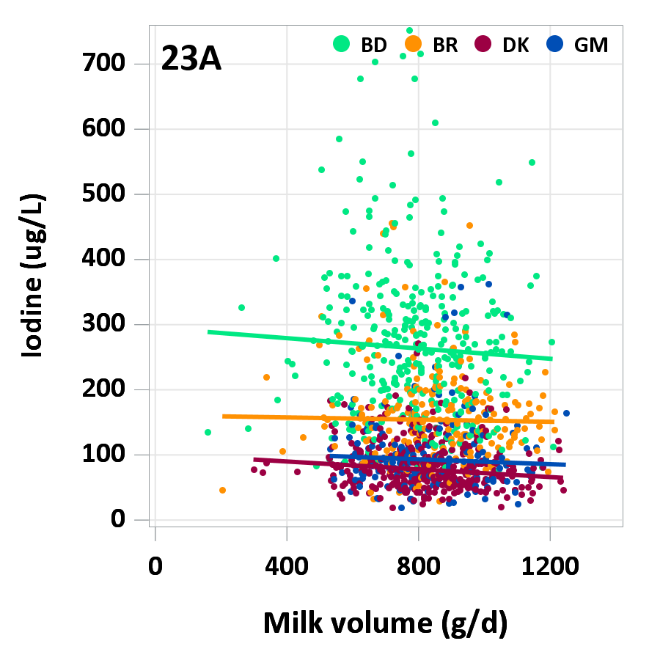** | **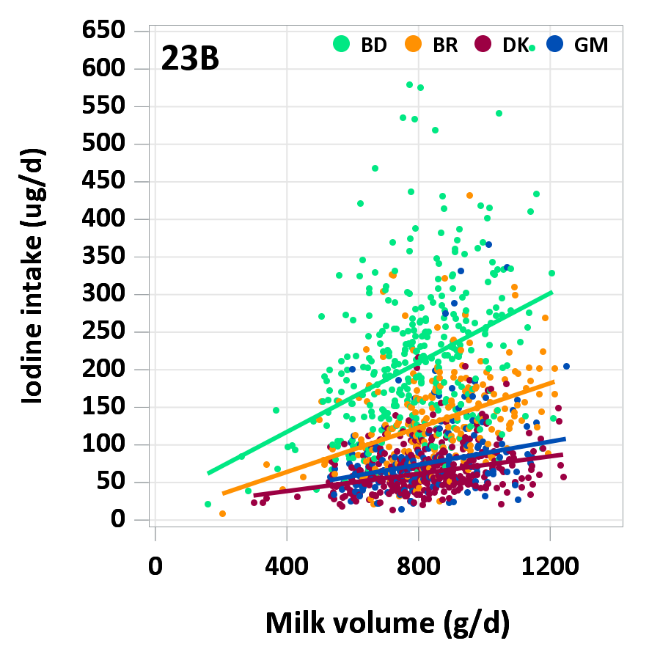** |
| --- | --- |

**Supplementary Figure 24. Vitamin A**

| **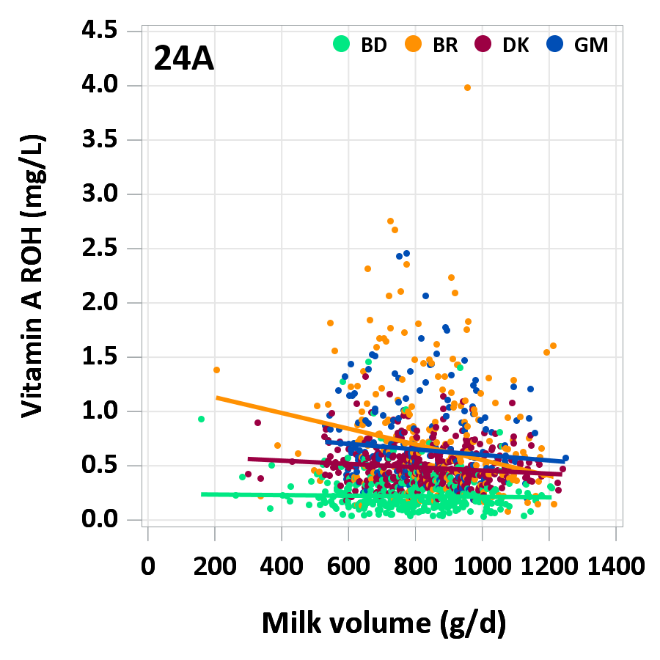** | **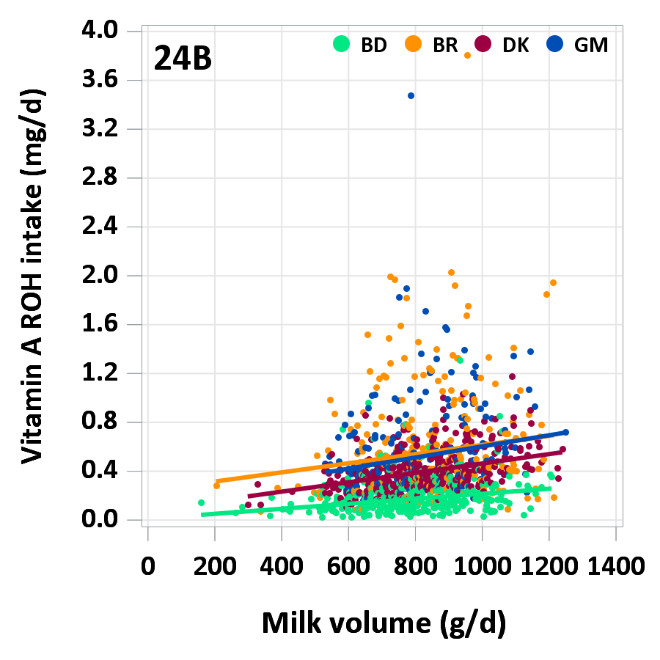** |
| --- | --- |

**Supplementary Figure 25. Gamma tocopherol**

| **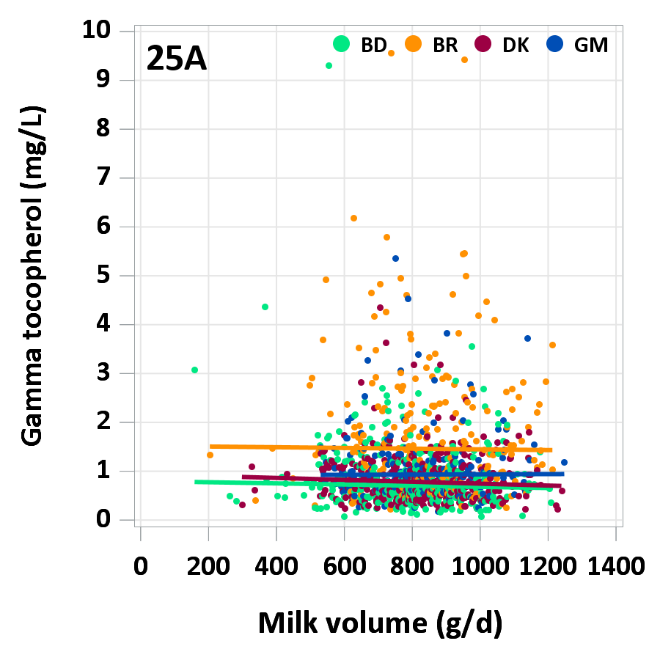** | **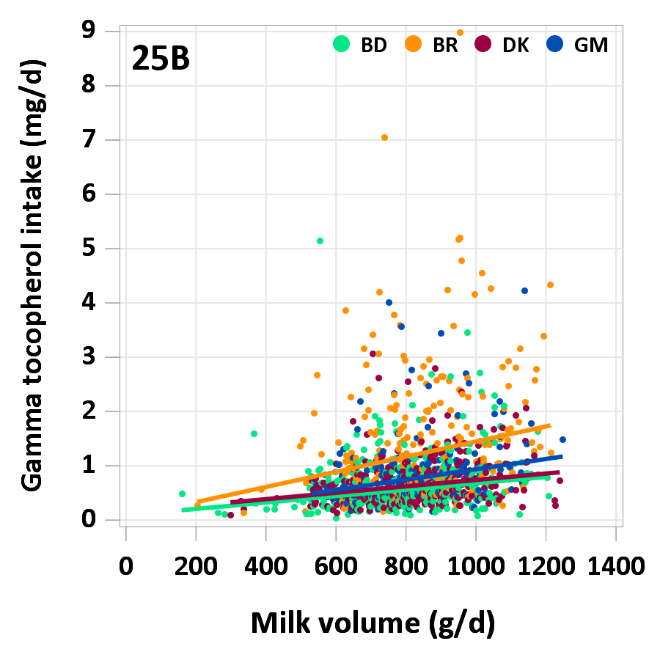** |
| --- | --- |

**Supplementary Figure 26. Alpha tocopherol**

| **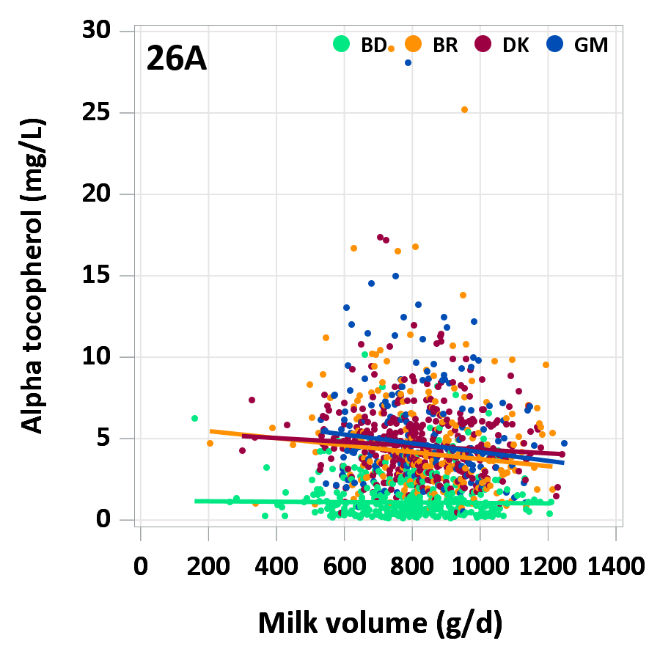** | **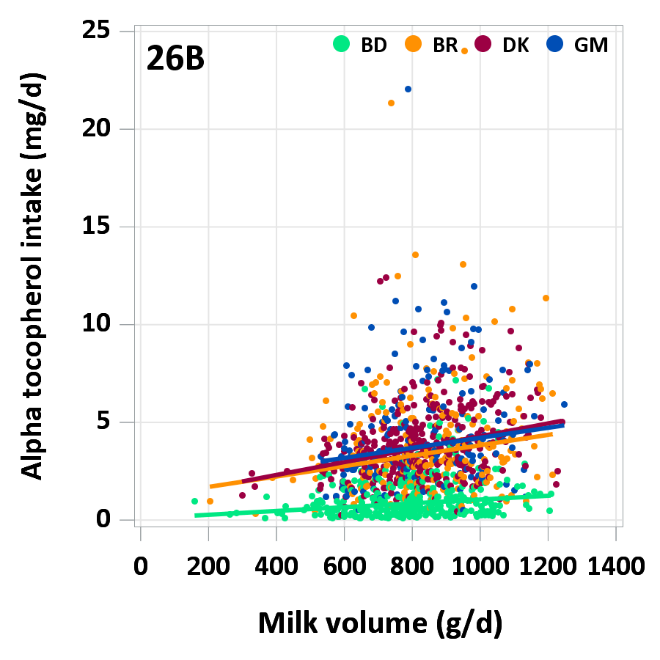** |
| --- | --- |

**Supplementary Figure 27. Vitamin D anti-rachitic activity**

| **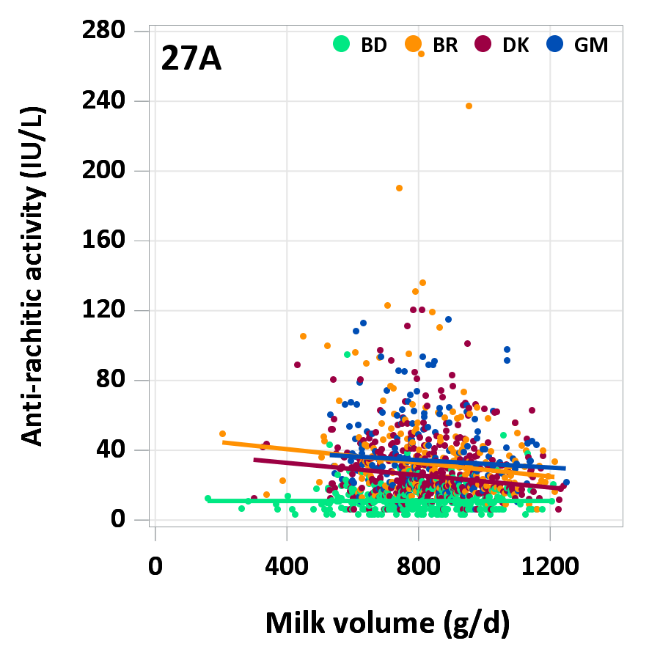** | **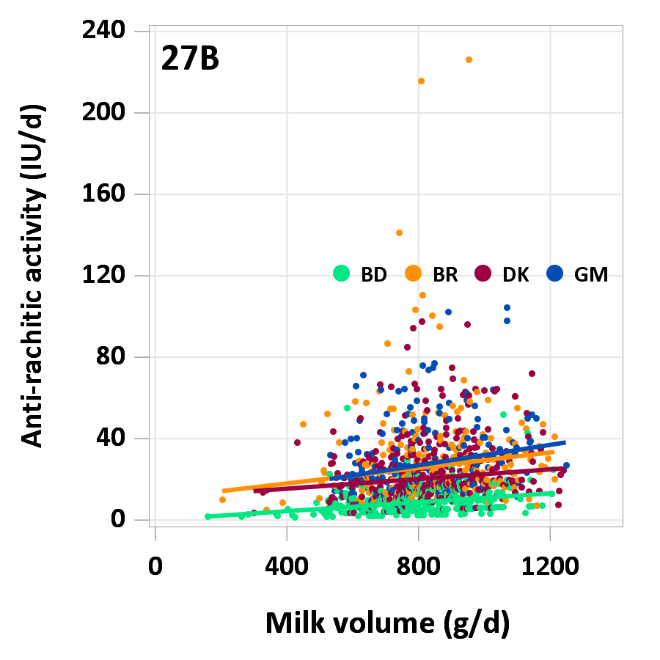** |
| --- | --- |
